# SCF^SLF^-mediated cytosolic degradation of S-RNase is required for cross-pollen compatibility in S-RNase-based self-incompatibility in *Petunia hybrida*

**DOI:** 10.3389/fgene.2014.00228

**Published:** 2014-07-22

**Authors:** Wei Liu, Jiangbo Fan, Junhui Li, Yanzhai Song, Qun Li, Yu'e Zhang, Yongbiao Xue

**Affiliations:** ^1^State Key Laboratory of Molecular Developmental Biology, Institute of Genetics and Developmental Biology, Chinese Academy of Sciences and National Center for Plant Gene ResearchBeijing, China; ^2^University of Chinese Academy of SciencesBeijing, China

**Keywords:** self-incompatibility, self-pollen incompatibility, cross-pollen compatibility, S-RNase localization, SCF^SLF^, ubiquitin/26S proteasome system

## Abstract

Many flowering plants adopt self-incompatibility (SI) to maintain their genetic diversity. In species of Solanaceae, Plantaginaceae, and Rosaceae, SI is genetically controlled by a single *S*-locus with multiple haplotypes. The *S*-locus has been shown to encode S-RNases expressed in pistil and multiple SLF (*S*-locus F-box) proteins in pollen controlling the female and male specificity of SI, respectively. S-RNases appear to function as a cytotoxin to reject self-pollen. In addition, SLFs have been shown to form SCF (SKP1/Cullin1/F-box) complexes to serve as putative E3 ubiquitin ligase to interact with S-RNases. Previously, two different mechanisms, the S-RNase degradation and the S-RNase compartmentalization, have been proposed as the restriction mechanisms of S-RNase cytotoxicity allowing compatible pollination. In this study, we have provided several lines of evidence in support of the S-RNase degradation mechanism by a combination of cellular, biochemical and molecular biology approaches. First, both immunogold labeling and subcellular fractionation assays showed that two key pollen SI factors, PhS_3L_-SLF1 and PhSSK1 (SLF-interacting SKP1-like1) from *Petunia hybrida*, a Solanaceous species, are co-localized in cytosols of both pollen grains and tubes. Second, PhS_3L_-RNases are mainly detected in the cytosols of both self and non-self-pollen tubes after pollination. Third, we found that PhS-RNases selectively interact with PhSLFs by yeast two-hybrid and co-immunoprecipitation assays. Fourth, S-RNases are specifically degraded in compatible pollen tubes by non-self SLF action. Taken together, our results demonstrate that SCF^SLF-mediated^ non-self S-RNase degradation occurs in the cytosol of pollen tube through the ubiquitin/26S proteasome system serving as the major mechanism to neutralize S-RNase cytotoxicity during compatible pollination in *P. hybrida*.

## Introduction

Self-incompatibility (SI) is a mating strategy that allows individual of a species to discriminate self (genetically related) from non-self (genetically unrelated), and thus accept cross-pollen whereas reject self-one. These outcomes are referred to as cross-pollen compatibility (CPC) and self-pollen incompatibility (SPI), respectively. In many species of flowering plants, such discrimination is genetically controlled by a single *S*-locus with multiple haplotypes, self-pollen rejection occurs when the pollen *S* haplotype matches either of the two pistil *S* haplotypes and the *S*-locus has been shown to encode at least two components from both the pollen and pistil sides, controlling the male and female expression of SI specificity, respectively (De Nettancourt, 2001; Takayama and Isogai, 2005; Franklin-Tong, 2008; Zhang et al., 2009). In Solanaceae, Plantaginaceae and Rosaceae, the pistil *S* encodes a T2 family ribonuclease, named S-RNase (McClure et al., 1989; Clark et al., 1990; Lee et al., 1994; Murfett et al., 1994; Sassa et al., 1996; Xue et al., 1996), whereas the pollen *S* has been shown to encode multiple paralogous F-box proteins called the *S* Locus F-box (SLF) in Solanaceae and Plantaginaceae (Lai et al., 2002; Zhou et al., 2003; Sijacic et al., 2004; Qiao et al., 2004a; Kubo et al., 2010; Williams et al., 2014) and SLF (Entani et al., 2003) or *S*-haplotype-specific F-box protein (SFB) in Rosaceae (Ushijima et al., 2003; Yamane et al., 2003; Sassa et al., 2007). In addition, other factors, not encoded by the *S-locus*, have been found in pistil side and are involved at different stages of the SI reaction. So far, three essential pistil-modifier factors, 120K (120 kDa glycoprotein), HT-B protein and NaStEP (*Nicotiana alata* stigma expressed protein), have been identified in *Nicotiana* species (McClure et al., 1999; Hancock et al., 2005; Jimenez-Durán et al., 2013). 120K is a style-specific glycoprotein that is taken up by pollen tubes during pollination (Lind et al., 1996; Schultz et al., 1997). Suppression of 120K expression by RNAi prevents *S*-specific pollen rejection (Hancock et al., 2005). Immunolocalization shows that HT-B is also taken up by pollen tubes during pollination. Loss-of-function assays showed that HT-B is essential for pollen rejection (McClure et al., 1999; Kondo et al., 2002; O'Brien et al., 2002; Sassa and Hirano, 2006; Puerta et al., 2009). NaStEP is a Kunitz-type proteinase inhibitors and a positive regulator of HT-B stability in *Nicotiana alata* pollen tubes (Busot et al., 2008; Jimenez-Durán et al., 2013).

S-RNase is a pistil-specific glycoprotein and initially synthesized in transmitting cells of the style and then secreted into extracellular matrix of the transmitting tract tissue (Cornish et al., 1987; Anderson et al., 1989). S-RNase is very abundant and mainly found in the transmitting track of a mature style where the growth of self-pollen tube is arrested after pollination (Cornish et al., 1987; Xue et al., 1996). It is proposed that S-RNase likely functions as a cytotoxic ribonuclease to degrade RNA by gaining access to self-pollen tube whose growth is thus arrested, but non-self-pollen tube growth is unaffected (McClure et al., 1990; Luu et al., 2000; Liu et al., 2009). The S-RNase is necessary for the pistil to recognize and reject self-pollen (Huang et al., 1994; Lee et al., 1994; Murfett et al., 1994). Furthermore, the S-RNase alone determines the pistil specificity of SI (Karunanandaa et al., 1994).

The first pollen *S* determinant, *AhSLF-S_2_*, was identified as an F-box gene in *Antirrhinum hispanicum*, a member of Plantaginaceae (Lai et al., 2002). Most F-box proteins usually serve as a component of an SCF (SKP1/Cullin1/F-box) ubiquitin ligase complex that often results in its target substrate polyubiquitination and degradation (Smalle and Vierstra, 2004). Thus, the finding of AhSLF-S_2_ provided a clue to the biochemical mechanism of SI. It was proposed that its potential function is involved in the ubiquitin/26S proteasome system (UPS) (Lai et al., 2002). The UPS pathway uses ubiquitin-activating enzyme E1, ubiquitin-conjugating enzyme E2 and ubiquitin ligase E3 to catalyze substrate polyubiquitination for degradation by the UPS pathway (Bai et al., 1996). SCF complex is a class of multisubunit E3 ligase involved in recognition and polyubiquitination of specific target proteins for degradation by the UPS pathway (Cardozo and Pagano, 2004; Willems et al., 2004). For Cullin1, as a scaffold protein, its N-and C-terminal domains interact with SKP1 and Rbx1, respectively. The F-box domain of F-box proteins interacts with SKP1 and a separate domain with their specific substrates (Zheng et al., 2002). Importantly, Qiao et al. (2004a) have shown that this C-terminal region of AhSLF_2_ interacts with both its cognate self and non-self S-RNases. In addition, SSK1 (SLF-interacting SKP1-like 1) proteins have been shown to be a novel class of pollen-specific SKP1-like proteins that bridge Cullin1 and SLF proteins to form an SCF^SLF^ complex in *A. hispanicum* (Plantaginaceae), *Petunia* (Solanaceae) as well as in both *Prunus* and *Pyrus* (Rosaceae) (Huang et al., 2006; Zhao et al., 2010; Matsumoto et al., 2012; Xu et al., 2013; Entani et al., 2014; Li et al., 2014; Yuan et al., 2014). Taken together, these results showed that both SLF and SSK1 are components of an SCF complex. In addition, Cullin1 has been shown to be involved in both SI and UI (unilateral incompatibility) in Solanum (Li and Chetelat, 2010, 2013). Thus, an S-RNase degradation model has been proposed to explain the biochemical mechanism of S-RNase-based SI. The model posits that non-self S-RNases are degraded via the UPS pathway mediated by SCF^SLF^ complex in cross pollen tubes so that S-RNase cytotoxicity is restricted, whereas self S-RNase is somehow able to escape degradation to exert its cytotoxicity to pollen tubes (Qiao et al., 2004a; Hua and Kao, 2006). By contrast, the S-RNase compartmentalization model also has been proposed for the S-RNase restriction mechanism (Goldraij et al., 2006; McClure, 2009; McClure et al., 2011). This model posits that the majority of S-RNases are sequestered in vacuoles of pollen tube with a minority entering the cytosols to be recognized by SLF. Sequestered S-RNases are thus spatially separated from cellular RNAs. Self-recognition is hypothesized to release S-RNases from vacuoles and subsequently to inhibit self-pollen tube growth, whereas cross recognition would stabilize vacuoles to continue to sequester S-RNases. Therefore, it remains unclear how the cytotoxic effect of S-RNase is specifically restricted in compatible pollination.

To address this issue, in this study, we determined the subcellular location of two key pollen SI factors, PhS_3L_-SLF1 and PhSSK1, as well as of the pistil factor PhS_3L_-RNase in pollen tubes after pollination in *P. hybrida*. Our results revealed that PhS_3L_-SLF1 and PhSSK1 are localized in cytosols of pollen grains and tubes where the majority of S-RNases are also located in the cytosols after pollination. In addition, we found that PhSLFs interact with PhS-RNases. Furthermore, we found that non-self S-RNases are degraded through the SCF^SLF^-mediated UPS pathway in compatible pollen tubes. Taken together, our results demonstrate that the SCF^SLF^-mediated cytosolic degradation of S-RNase serves as the major mechanism to restrict its cytotoxicity to cross-pollen tubes in *P. hybrida*, providing an important insight into the biochemical and cellular mechanisms of S-RNase-based SI.

## Results

### Identification of the pollen-*S* genes of *P. hybrida*

To identify the pollen-*S* genes in *P. hybrida*, we first cloned four *PhSLF* alleles by a homology-based method from self-incompatible *S_1_S_1_*, *S_3_S_3_*, *S_3L_S_3L_*, and *S_v_S_v_* homozygous plants as described (Clark et al., 1990; Robbins et al., 2000; Qiao et al., 2004b). PhS_1_-SLF1 (GenBank accession number GQ121443.1), PhS_3_A-SLF1 (GenBank accession number AY639403.1), PhS_3L_-SLF1 (GenBank accession number GQ121445.1) and PhS_v_-SLF (GenBank accession number GQ121446.1) were found to belong to Type-1 SLFs (Supplementary Figures S1A,B) based on the classification by Kubo et al. (2010). We then isolated a promoter fragment derived from a 2120 bp sequence upstream of a pollen-specific gene *PhS_3_A-SLF1*. A binary vector *pBI101* containing the *PhS_3_A-SLF1* promoter fragment fused with a downstream GUS reporter gene (Supplementary Figure S2A) was introduced into self-incompatible lines of *S_1_S_v_* and *S_3_S_3L_* haplotypes, respectively. GUS activity analysis of the transgenic plants and wild-type revealed that this putative promoter fragment was sufficient to drive the GUS expression specifically in the anther (Supplementary Figure S2B), resulted from its expression in the pollen grains (Supplementary Figure S2C). To determine whether the *PhSLF* alleles encode the pollen *S*-determinants, we generated transgenic plants by introducing three alleles, *PhS_1_-SLF1*, *PhS_3L_-SLF1*, and *PhS_v_-SLF1* driven by the *PhS_3_A-SLF1* promoter into different self-incompatible lines of *S* haplotypes, respectively. Competitive interaction, where expression of two heteroallelic pollen-*S* genes in the same pollen grain causes breakdown of pollen self-incompatibility, has been used to test the validity of the pollen-*S* genes (Lewis, 1947; Sijacic et al., 2004; Qiao et al., 2004b). The *PhS_3L_-SLF1* transgene caused breakdown of self-incompatibility in *S_3_S_3L_* but not *S_3L_S_v_* haplotypes (Supplementary Figures S3A,B, Supplementary Tables S1,S2), indicating that it functions as the pollen-*S* product against non-self S_3_-RNase. Consistent with this result, all progeny from self-pollination of the primary *S_3_S_3L_*/*PhS_3L_-SLF1* plants inherited *S_3_-RNase* and the transgene, with an *S_3_S_3_* to *S_3_S_3L_* segregation ratio of ca.1:1 as predicted (Supplementary Figure S4A, Supplementary Table S2). We also examined the inheritance of the *PhS_3L_-SLF1* transgene and progeny *S*-genotypes from pollination of wild-type *S_3_S_3L_* plants with the pollen from the *S_3_S_3L_*/*PhS_3L_-SLF1*-3 transgenic plants. All progeny also inherited *S_3_-RNase* and the transgene, with an *S_3_S_3_* to *S_3_S_3L_* segregation ratio of ca.1:1 as predicted (Supplementary Figure S4A). Furthermore, the *S_1_S_v_*/*PhS_3L_-SLF1::FLAG* plants also caused self-incompatibility breakdown (Supplementary Figure S3C, Supplementary Table S3). All progeny from self-pollination of the primary *S_1_S_v_*/*PhS_3L_-SLF1::FLAG* plants inherited *S_1_-RNase* and the transgene, with an *S_1_S_1_* to *S_1_S_v_* segregation ratio of ca.1:1 as predicted (Supplementary Figure S4B, Supplementary Table S3). Nevertheless, the *PhS_1_-SLF1* transgene did not show a competitive interaction in the *S_1_S_v_* transgenic plants (Supplementary Figure S3D, Supplementary Table S4), and the *PhS_v_-SLF1* transgene did not show a competitive interaction in the *S_3L_S_v_* transgenic plants either (Supplementary Figure S3E, Supplementary Table S5), showing that the *PhS_1_-SLF1* and *PhS_v_-SLF1* do not function as the pollen-*S* at least against S_v_-RNase and S_3L_-RNase, respectively. Taken together, these results showed that the *PhS_3L_-SLF1* and *PhS_3L_-SLF1::FLAG* act as the functional pollen-*S* against *S_3_-RNase* and *S_1_-RNase*, respectively.

### PhS_3l_-SLF1 and PhSSK1 both localize to the cytosols of pollen grains and pollen tubes

**Figure 1:**
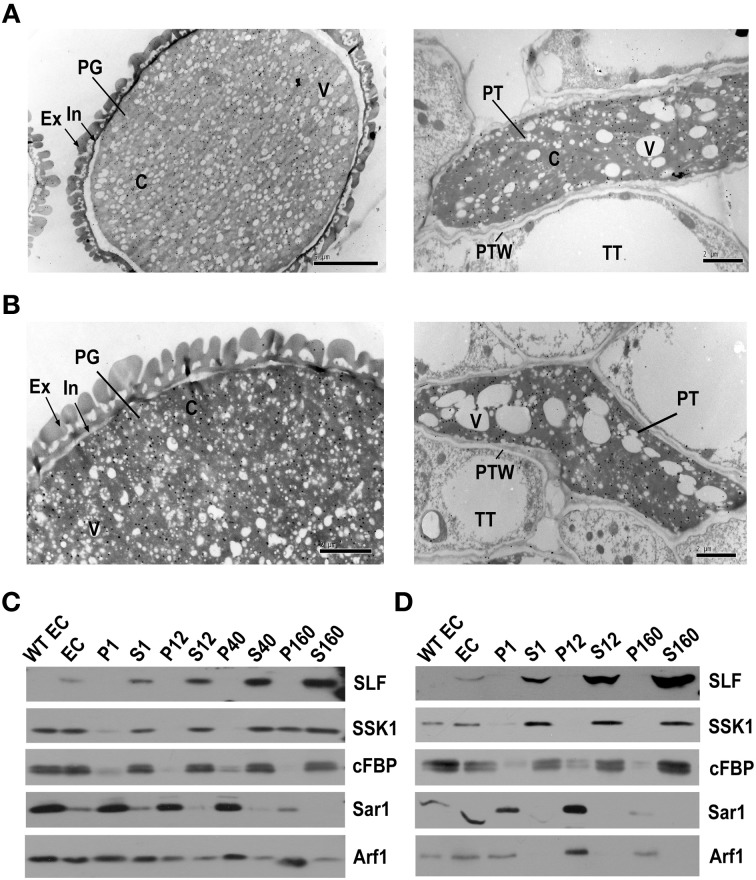
**PhS_3L_-SLF1 and PhSSK1 are both located in the cytosol of pollen grains and pollen tubes. (A)** Immunogold labeling of PhS_3L_-SLF1::FLAG in pollen grain (left) and pollen tube (right). **(B)** Immunogold labeling of PhSSK1 in pollen grain (left) and pollen tube (right). PG, pollen grain; Ex, exine; In, intine (arrow); C, cytosol; V, vacuole; PT, pollen tube; PTW, pollen tube wall; TT, transmitting tract tissue. **(C)** Western blot detection of PhS_3L_-SLF1::FLAG (SLF) and PhSSK1 (SSK1) in subcellular fractions of pollen grains. **(D)** Western blot detection of PhS_3L_-SLF1::FLAG (SLF) and PhSSK1 (SSK1) in subcellular fractions of *in vitro* germinated pollen tubes. WT EC and EC denote entire cell homogenates from wild-type and the transgenic pollen grains or pollen tubes, respectively. Pellet fractions (P1, P12, P40, and P160) and supernatant fractions (S1, S12, S40, and S160) are derived from differential centrifugation at 1000, 12,000, 40,000, and 160,000 g, respectively. cFBP, Sar1, and Arf1 are marker antibodies for cytosol, endoplasmic reticulum (ER) and Golgi, respectively.

To examine the localization of the pollen SI factors PhS_3L_-SLF1 and PhSSK1, we performed immunogold labeling and subcellular fractionation experiments. For immunogold labeling experiment of PhS_3L_-SLF1, we used FLAG antibody to detect its expression in the mature pollen grains and pollinated pollen tubes of the *S_1_S_v_*/*PhS_3L_-SLF1::FLAG* transgenic plants exhibiting the competitive interaction described above. The FLAG gold particles corresponding to PhS_3L_-SLF1::FLAG are distributed in the cytosols of both pollen grains and pollen tubes rather than associated with any discernible organelle (Figure 1A). This finding is similar to that found for AhSLF-S_2_ in *A. hispanicum* (Wang and Xue, 2005). As controls, the FLAG antibody did not label any signal in both pollen grain, pollen tube and pollinated style of wild-type (Supplementary Figure S5A), confirming the specificity of FLAG antibody.

To examine the subcellular location of PhSSK1, *P. hybrida* SLF-interacting SKP1-like1, an adaptor for an SCF^SLF^ complex, we used PhSSK1 antibody to detect its expression in the mature pollen grains and pollinated pollen tubes of wild-type plants. First, to examine the specificity of the PhSSK1 antibody, we extracted proteins from different tissues for western blot analysis and found strong signals at approximately 30 kDa in the pollen grains and pollen tube extracts, and no signal was found with the protein extracts from other organs, including leaf and pistil, indicating that the antibody is specific for its target protein. In addition, western blotting analysis using rabbit pre-immune serum for PhSSK1 antibody showed no apparent signal in the protein extracts from all of these organs examined (Supplementary Figure S5B). In addition, we further performed immunogold labeling using rabbit pre-immune serum as a negative control and the pre-immune serum did not label any signal in wild-type pollen grain and pollen tube as well as pollinated style (Supplementary Figure S5C). Thus, the PhSSK1 antibody has strong target specificity and we performed immunogold labeling using the antibody to detect the subcellular location of PhSSK1. The immunogold labeling results showed that the PhSSK1 gold particles exhibited a similar distribution to the PhS_3L_-SLF1::FLAG in the cytosols of both pollen grains and tubes (Figure 1B). Taken together, these results indicated that the pollen SI factors PhS_3L_-SLF1 and PhSSK1 are both localized in the cytosols of the pollen grains and pollinated pollen tubes.

Nevertheless, because it is hard to clearly distinguish the endomembrane system from the cytosols in our immunogold labeled sections, we further carried out subcellular fractionation experiments. The PhS_3L_-SLF1::FLAG (SLF) was always co-purified with the supernatant fractions and significantly enriched in the S160 fraction, representing the cytosolic fraction (Cherry, 1974; Quail, 1979; Arrese et al., 2001; Agrawal et al., 2011; Rangel et al., 2013) of pollen grains (Figure 1C) and pollen tubes (Figure 1D), consistent with the immunogold labeling results. PhS_3L_-SLF1::FLAG specific signal detected in pollen grains and pollen tubes of transgenic plant, but not in wild-type pollen grains and pollen tubes (Figures 1C,D), indicating that FLAG antibody is specific for its target protein. In addition, subcellular fractionation experiment further showed a similar location for PhSSK1 (SSK1) (Figures 1C,D). Interestingly, SSK1 showed a dynamic localization seen in P160 fractions of the pollen grains (Figure 1C), similar to the Golgi marker Arf1 observed previously (Pertl et al., 2009) though it was not clear how this occurs. Taken together, these results showed that the pollen factors PhS_3L_-SLF1 and PhSSK1 are located in the cytosols of the pollen grains and pollen tubes.

### S-RNases are predominantly localized in the cytosols of both compatible and incompatible pollen tubes

Previous studies have shown that S-RNases appear to have different locations. Luu et al. found that they are located in the cytosols of pollen tubes in *Solanum chacoense* by immunocytochemical labeling (Luu et al., 2000). Whereas Goldraij et al. (2006) showed that they are largely compartmentalized in vacuoles in *Nicotiana* by immunolocalization. To examine the localization of PhS_3L_-RNase (GenBank accession number AJ271065.1), we performed both immunogold labeling and subcellular fractionation experiments. To examine the specificity of PhS-RNase antibody, proteins from different tissues were extracted and detected. After western blotting, strong signals were only found in the *S_3L_S_3L_* and *S_3_S_3_* style extracts, but no signal was found in pollen grains, pollen tubes, leaves, *S_1_S_1_* and *S_v_S_v_* styles. As a negative control, western blotting analysis using rabbit pre-immune serum for PhS-RNase antibody detected, no apparent signal in all of the organs examined (Supplementary Figure S5D). We further performed the immunogold labeling using the PhS-RNase antibody and rabbit pre-immune serum as negative controls. PhS-RNase antibody did not label any signal in pollen tube without style extract treatment (Supplementary Figure S5E) and the pre-immune serum also did not label any signal in pollinated pistil (Supplementary Figure S5F). To further confirm the PhS-RNase antibody specificity, total proteins of *S_3L_S_3L_* styles were isolated and analyzed by two-dimensional polyacrylamide gel and western blot. A strong signal was observed at approximately 30 kDa and pH 9.0 (Supplementary Figure S6A). Then the spot was excised from the gel and identified by liquid chromatography/tandem mass spectrometry (LC-MS/MS). In the MASCOT search, a total of 204 peptides matched the sequence of PhS_3L-RNase_ in total 900 peptides identified by the LC-MS/MS spectra, and matched peptides have a coverage 43% sequence of PhS_3L_-RNase (Supplementary Figures S6A,B). The other peptides were identified from the 30 kDa protein spot did not match PhS_3L_-RNase (Supplementary Table S6). In addition, other unidentified minor fragments were judged to be derived from contaminated and/or degraded proteins. Therefore, our proteomic data indicated that the 30 kDa protein represents PhS_3L_-RNase. Taken together, these results demonstrated that the PhS-RNase antibody specifically detects its target protein.

**Figure 2:**
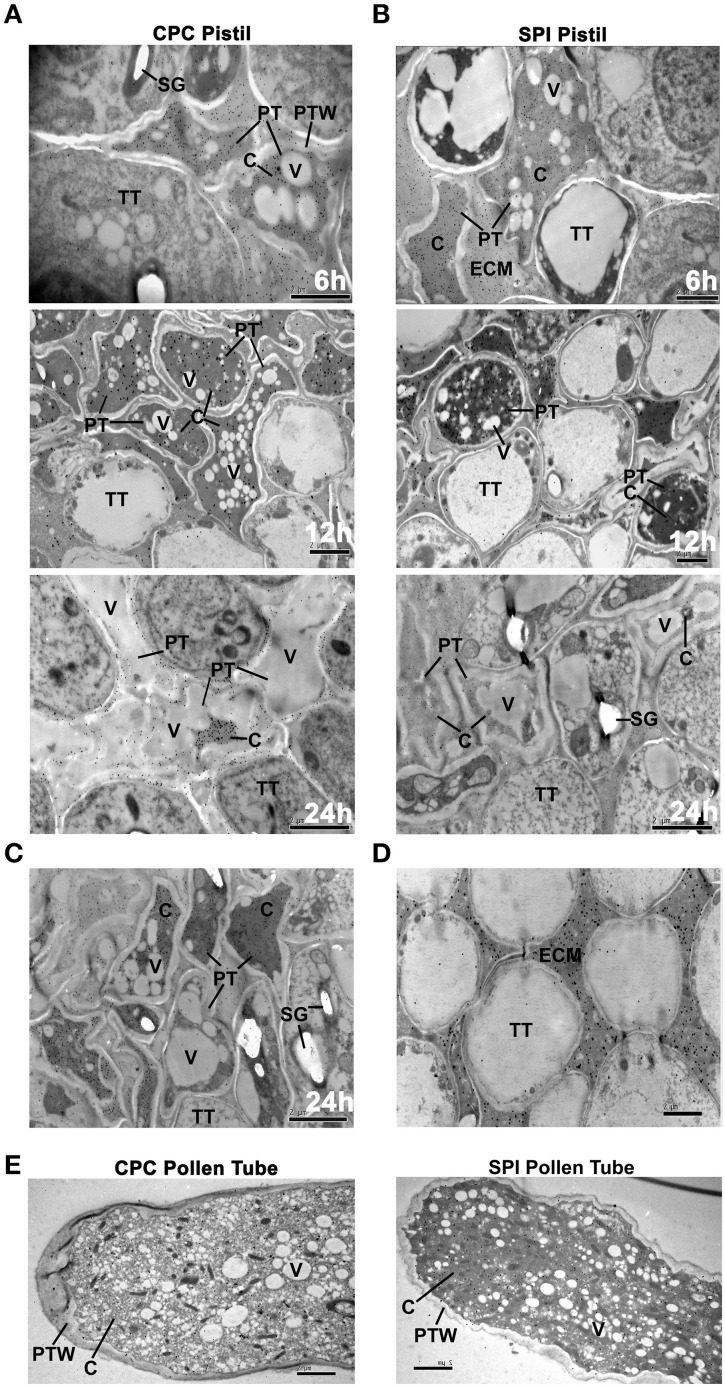
**S-RNases localize to the cytosol of both compatible and incompatible pollen tubes. (A)** Immunogold labeling of PhS_3L_-RNase in cross-sectioned pistils of 6, 12, and 24 h post compatible pollination (CPC), respectively. A zone that is relatively close to the pollen tube tip was observed. **(B)** Immunogold labeling of PhS_3L_-RNase in cross-sectioned pistils after 6, 12, and 24 h post incompatible pollination (SPI), respectively. A zone that is relatively close to the pollen tube tip was observed. **(C)** Immunogold labeling of PhS_3L_-RNase in cross-sectioned pistils after 24 h post compatible pollination. A zone that is relatively close to the upper one-third pollen tube was observed. **(D)** A region without pollen tube showing PhS_3L_-RNase accumulation mainly in ECM of a mature pistil as positive control. **(E)** Immunogold labeling of PhS_3L_-RNase in *in vitro* germinated pollen tube of CPC (*S_v_*) and SPI (*S_3L_*), respectively (longitudinal-section). PT, pollen tube; PTW, pollen tube wall; TT, transmitting tract tissue; C, cytosol; V, vacuole; SG, starch granule; ECM, extracellular matrix.

For immunogold labeling experiment of PhS_3L_-RNase, we used the PhS-RNase antibody to detect its expression in compatible (CPC) and incompatible (SPI) pollinated styles. Both CPC and SPI styles at 6, 12, and 24 h post-pollination were stained with aniline blue and monitored by a fluorescence microscope (Supplementary Figure S7), showing that the growth of incompatible pollen tubes is arrested around the upper one-third of the style and the compatible pollen tubes have reached the bottom of the style 24 h post-pollinations as described previously (Singh et al., 1992). The CPC and SPI styles of 6 h post-pollination were used to observe the stylar regions containing the apical tips of pollen tubes, where their elongations occurred. The subcellular localization of PhS_3L_-RNase was observed for the transverse sections of the CPC and SPI stigmas at 6 h post-pollination, the upper one-third of CPC and SPI styles at 12 h post-pollination, the bottom of styles at 24 h post compatible pollination and the upper one-third of styles at 24 h post incompatible pollination, respectively. The PhS_3L_-RNase gold particles were found to be evenly distributed in the cytosols of both CPC and SPI pollen tubes (relatively close to the pollen tube tip) growing in styles at 6, 12, and 24 h post-pollination, respectively, but not found in vacuoles and any other discernible organelles (Figures 2A,B). In particular, the gold particles were not found in vacuoles when significant cytosol vacuolization occurred in pollen tubes growing in style around 24 h post compatible pollination. It is noteworthy that there are still some gold particles in the pollen tubes within the style around 24 h post compatible pollination (Figure 2A). In addition, a distal zone from the pollen tube tip (upper one-third) of CPC after 24 h also were observed by immunogold labeling, and this result showed that the PhS_3L_-RNase gold particles also are found to be evenly distributed in the cytosols but not found in vacuoles of 24 h post compatible pollination (Figure 2C). A region without pollen tubes showing PhS_3L_-RNase accumulation occurred mainly in extracellular matrix (ECM) of a mature pistil as a positive control (Figure 2D). Taken together, these results indicated that PhS_3L_-RNases are predominantly localized in the cytosols of the pollen tubes in styles of early, middle, and late stages after both CPC and SPI pollinations.

To further examine the subcellular localization of PhS_3L_-RNase, we carried out a similar immunogold labeling experiment using an *in vitro* pollen germination system. In order to test the validity of *in vitro* pollen germination system in *P. hybrida*, we carried out the following experiments. Often, compatible pollen tubes grow about 2 cm in pistil at 24 h post-pollination in *P. hybaida*. Pollen tubes also can grow about 2 cm in *in vitro* pollen germination medium after 24 h (Supplementary Figure S8A), indicating that pollen tube growth *in vitro* system is largely similar to that *in vivo* pollination reaction. In addition, the *S_v_* and *S_3L_* pollen tubes were continuously challenged with *S_3L_S_3L_* style extracts to mimic the CPC and SPI pollinations, respectively, both CPC and SPI pollen tube growth was inhibited compared to controls, but the SPI pollen tubes showed a more significant growth inhibition compared with CPC pollen tubes (Supplementary Figures S8B,C, Supplementary Table S7). Nevertheless, the control pollen tubes without stylar extracts showed much better growth compared with both CPC and SPI ones, suggesting that the extracts contained a strong inhibitory activity of pollen tube growth. Further work is needed to address this issue. The results showed that *in vitro* (5 h) compatible pollen tubes are about 0.02 cm, and incompatible about 0.017 cm (Supplementary Figures S8B,C), also similar to that of compatible and incompatible tubes (6 h) *in vivo* (Supplementary Figure S7). Taken together, these results showed that the *in vitro* pollen germination system in *P. hybrida* (Supplementary Figure S8) are similar to that by Qiao et al. (2004a) in *Antirrhinum* and Meng et al. (2014) in apple. The *in vitro* pollen germination system can exclude a high background interference of S-RNases in style. Although the style extracts *in vitro* appeared to lose their activity after long time incubation, this system can mimic the CPC and SPI reaction for at least first several hours of the reaction. Using the *in vitro* pollen germination system, we further examined the subcellular localization of PhS_3L_-RNase in pollen tubes, and the immunogold labeling results showed that the PhS_3L_-RNase gold particles were found to be distributed in the cytosols of both CPC and SPI pollen tubes (Figure 2E). Taken together, these results showed that PhS_3L_-RNases are localized in the cytosols of the CPC and SPI pollen tubes in the *in vitro* pollen germination system, similar to that found in *in vivo* (Figures 2A,B).

For detection of smaller membranous structures such as endosomes and prevacuolar compartment (PVC), immunogold labeling is not feasible because of the size of commercial colloidal gold is 5–20 nm, mostly 10 nm for transmission electron microscope (TEM), which is beyond the thickness of biomembrane. In addition, it is clearly seen that immunogold particles are not present in the vacuoles, starch granules and mitochondria structures from the images of S-RNases immunogold labeling in pollen tubes (Figures 2A–C). Both vacuoles and starch granules are electron-luculent in TEM pictures. The edge of vacuoles is clearer and smooth in pollen tube. However, the starch granules are more dim and irregular. To exclude the possibility of endosome, endoplasmic reticulum (ER), Golgi and PVC localization of S-RNase, we carried out subcellular fractionation experiments using the pulse challenged pollen tubes of the *in vitro* pollen germination system. Both *S_v_* (CPC) and *S_3L_* (SPI) pollen tubes were treated with *S_3L_S_3L_* style extracts as described above. The western blot results showed that the overwhelming majority of PhS_3L_-RNase was always co-purified with the supernatant fractions and significantly enriched in the S160 fractions of both CPC and SPI pollen tubes (Supplementary Figure S9A), representing the cytosolic fractions (Cherry, 1974; Quail, 1979; Arrese et al., 2001; Agrawal et al., 2011; Rangel et al., 2013), indicating that PhS-RNases have a predominant cytosolic location but a minor amount associated with a microsome-like fraction. Taken together, these results demonstrated that PhS_3L_-RNases are predominantly localized in the cytosols of the CPC and SPI pollen tubes, similar to that found in *S. chacoense* (Luu et al., 2000).

### PhSLF interacts with PhS-RNase in both yeast and pollen tubes

**Figure 3:**
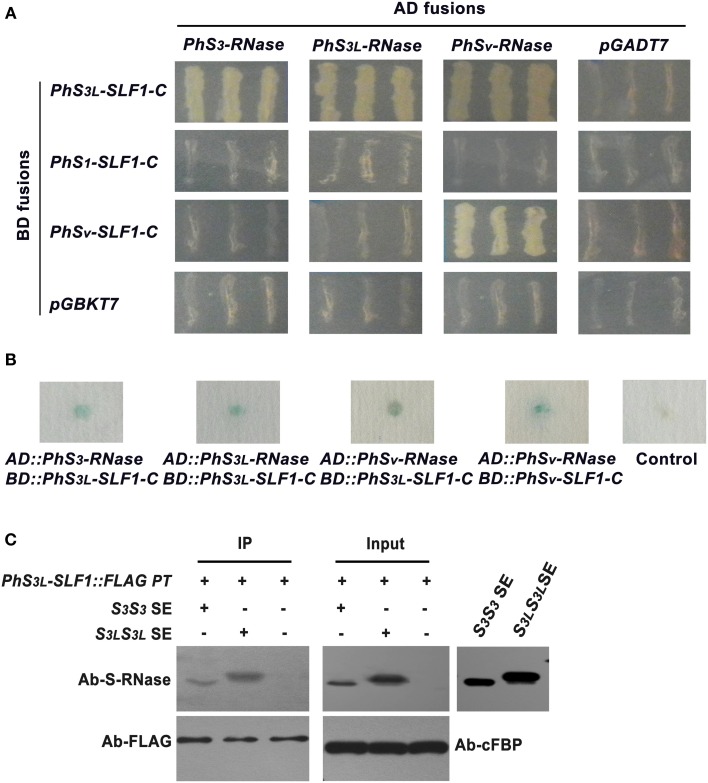
**PhSLFs selectively interact with PhS-RNases. (A)** Yeast two-hybrid assays between PhSLFs and PhS-RNases. Cells of yeast strain AH109 containing various combinations of bait (BD fusions) and prey (AD fusions) were tested for their growth on selective medium SD/-Ade-His-Leu-Trp. The empty vectors *pGBKT7* and *pGADT7* were negative controls. **(B)** The β-galactosidase activity assay was used to further test the interaction of PhSLFs and PhS-RNases. The combination of empty *pGBKT7* and *pGADT7* was used as a negative control. **(C)** Co-immunoprecipitation assays between PhS_3L_-SLF1::FLAG and PhS_3_-RNase or PhS_3L_-RNase using *in vitro* pollen germination system. Untreated pollen tubes were used as negative control (CK). Samples from SPI (treated with *S_3_S_3_* SE), CPC (treated with *S_3L_S_3L_*SE) and CK pollen tubes were immunoprecipitated by FLAG antibody and detected by PhS-RNase antibody and FLAG antibody, respectively. Challenged but not immmunoprecipitated SPI, CPC, and CK pollen tube samples were loaded as input. cFBP was detected as input loading control. PT, pollen tube; SE, style extract.

Although our results have shown that PhS_3L_-RNase, PhS_3L_-SLF1, and PhSSK1 are all localized to the cytosol of pollen tubes, it remained unclear whether an interaction between PhSLFs and PhS-RNases occurs. To examine the physical interaction between PhSLFs and PhS-RNases, we used a yeast two-hybrid assay. The C-terminal coding sequences of *PhS_3L_-SLF1*, *PhS_1_-SLF1*, and *PhS_v_-SLF1* (after removal of F-box domain) were introduced into *pGBKT7* vector and expressed as a fusion to GAL4 DNA binding domain (BD), whereas the full-length coding sequences of *PhS_3L_-RNase*, *PhS_3_-RNase* (GenBank accession number U07363.1) and *PhS_v_-RNase* (GenBank accession number AJ271062.1) were introduced into *pGADT7* vector and expressed as a fusion to transcriptional activating domain (AD). Yeast cells co-transformed with the *PhS_3L_-SLF1-C* and the *PhS_3_-RNase*, *PhS_3L_-RNase* or *PhS_v_-RNase* constructs grew well on both Leu-/Trp- and Leu-/Trp-/His-/Ade-media, showing that a physical interaction had occurred between PhS_3L_-SLF1-C and *PhS_3_-RNase*, *PhS_3L_-RNase* or *PhS_v_-RNase* in yeast. In addition, the C-terminal region of PhS_v_-SLF1 only interacts with PhS_v_-RNase, rather than the PhS_3_-RNase and PhS_3L_-RNase. We did not detect interactions between the C-terminal region of PhS_1_-SLF1 and PhS_3_-RNase, PhS_3L_-RNase or PhS_v_-RNase. Yeast transformed with the negative control plasmids *AD::PhS-RNases* and *pGBKT7* or *BD::PhSLFs* and *pGADT7* did not grow (Figure 3A). Furthermore, the β-galactosidase reporter activity was detected and appeared to be positive in yeast cells co-transformed with *BD::PhSLFs-C* and *AD::PhS-RNases* (Figure 3B). Taken together, these results show that PhSLFs selectively interact with PhS-RNases in yeast cells.

To confirm the interactions between the PhSLF and PhS-RNase, we performed a co-immunoprecipitation experiment. To examine the co-immunoprecipitation specificity of FLAG antibody, *in vitro* germinated *S_1_*/*PhS_3L_-SLF1::FLAG* pollen tubes from selfed progeny of the primary *S_1_S_v_*/*PhS_3L_-SLF1::FLAG* plants described above were continuously treated with *S_3L_S_3L_* style extracts, whereas wild-type *S_1_* and *S_3L_* pollen tubes were continuously challenged with *S_3L_S_3L_* style extracts were used as negative controls, respectively. Equal amounts samples were immunoprecipitated by FLAG antibody and detected by PhS-RNase antibody. Western blot results showed that PhS_3L_-RNase detected in the sample of *PhS_3L_-SLF1::FLAG* pollen tubes, but no apparent signal was found in wild-type *S_1_* and *S_3L_* pollen tubes (Supplementary Figure S10). Thus, the FLAG antibody has a strong target specificity and we thus performed co-immunoprecipitation experiment using the antibody to detect the interactions between PhS_3L_-SLF1 and PhS_3_-RNase or PhS_3L_-RNase. *In vitro* germinated *S_1_/PhS_3L_-SLF1::FLAG* pollen tubes were continuously treated with *S_3_S_3_* and *S_3L_S_3L_* style extracts to mimic CPC and SPI responses, respectively. Untreated pollen tubes were used as negative control. Equal amounts of the treated and untreated pollen tube samples were immunoprecipitated by FLAG antibody and detected by both PhS-RNase and FLAG antibodies, respectively. Western blot results showed that the specific proteins, similar to that detected in the wild-type *S_3_S_3_* and *S_3L_S_3L_* style extracts, were also detected in the treated pollen tubes by the PhS-RNase antibody, but not detected when using the untreated pollen tubes (Figure 3C), showing that PhS_3_-RNase and PhS_3L_-RNase were specifically precipitated by the anti-FLAG antibody in both CPC and SPI responses. Taken together, these results showed that the PhS_3L_-SLF1 interacts with PhS_3_-RNase and PhS_3L_-RNase.

### PhS_3L_-RNases are polyubiquitinated in cross-compatible pollen tubes

**Figure 4:**
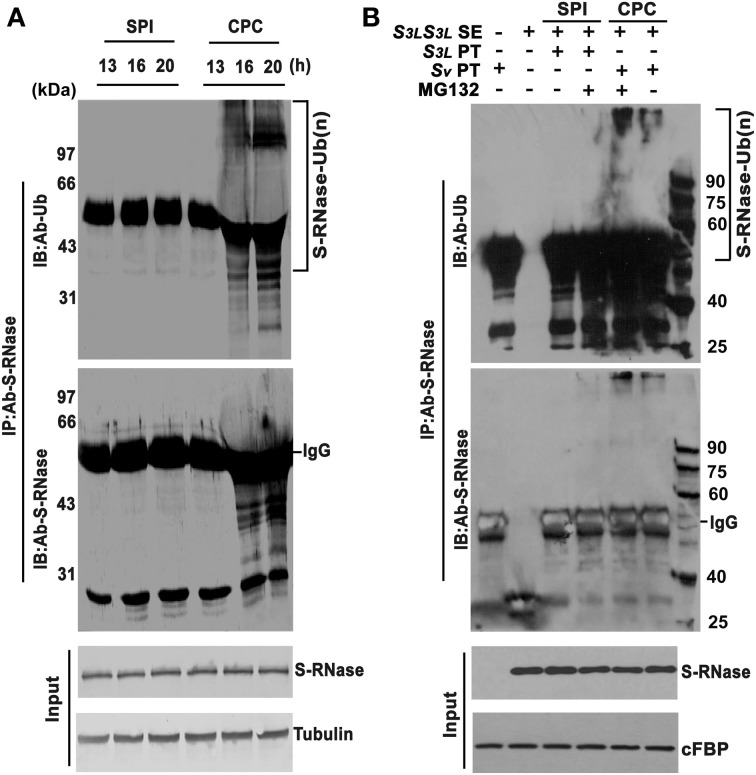
**S-RNases are polyubiquitinated in compatible pollen tubes. (A)** PhS-RNases are polyubiquitinated in pollen tubes of compatible pollination. Pollen tubes were dissected from styles after 13, 16, and 20 h post-pollination. Samples extracted and immunoprecipitated by PhS-RNase antibody and detected by ubiquitin (Ub) antibody and PhS-RNase antibody. PhS-RNase and tubulin were detected as input and loading control. **(B)** PhS-RNases are polyubiquitinated in CPC response in *in vitro* pollen germination system. *In vitro* germinated *S_3L_* (SPI) and *S_v_* (CPC) pollen tubes challenged by *S_3L_S_3L_* style extract were subjected to MG132 treatment (+) or not (−). Samples of SPI, CPC, and CK were immunoprecipitated by PhS-RNase antibody and detected by ubiquitin (Ub) antibody and PhS-RNase antibody, respectively. Style extracts and immunoprecipitation of the untreated pollen tubes samples were used as negative controls. Challenged but not immmunoprecipitated pollen tube samples were loaded as input. PhS_3L_-RNase and cFBP were detected as input and loading control. S-RNase-Ub (n) denotes polyubiquitinated S-RNase. IgG indicates heavy chain. Molecular weights in kilodalton (kDa) are shown on right or left side of the blots.

Previously, AhS-RNases have been shown to be polyubiquitinated in *A. hispanicum in vivo* (Qiao et al., 2004a). To examine whether PhS-RNases were polyubiquitinated in compatible pollination *in vivo*, *S_3L_S_3L_* styles were pollinated with *S_3L_* (SPI) and *S_v_* (CPC) pollen, respectively. Because S-RNase is highly abundant in the style (Roalson and McCubbin, 2003), it is difficult to detect any difference of ubiquitinated S-RNase between compatible and incompatible pollen tubes within the pollinated styles due to a high background. To overcome the potential problems, the CPC and SPI pollen tubes were dissected out from styles of 13, 16, and 20 h post-pollination for lysis and protein extraction. The dissected pollen tube samples are enriched for pollen tubes (Supplementary Figure S11). Ubiquitination was examined by co-immunoprecipitation using PhS-RNase antibody. Western blot signals of polyubiquitinated PhS_3L_-RNases were specifically detected in the CPC pollen tube samples after 16 and 20 h post-pollination by both PhS-RNase and ubiquitin antibodies, but little in the SPI samples (Figure 4A). Thus, these results indicated that PhS_3L_-RNases are polyubiquitinated in compatible pollen tubes in *P. hybrida in vivo*.

To confirm this finding, we examined PhS_3L_-RNase polyubiquitination using the *in vitro* pollen germination system as described. Both *S_3L_* (SPI) and *S_v_* (CPC) pollen tubes were challenged with *S_3L_S_3L_* style extracts in conjunction with or without MG132 (a peptide aldehyde effectively blocking the proteolytic activity of the 26S proteasome complex) treatment, respectively. The challenged *S_v_* and *S_3L_* pollen tube samples were immunoprecipitated using PhS-RNase antibody and detected by both PhS-RNase and ubiquitin antibodies, respectively. Polyubiquitinated PhS_3L_-RNase was predominantly detected in the CPC pollen tube samples, but not in the SPI samples (Figure 4B). In addition, polyubiquitinated PhS_3L_-RNases were accumulated to a higher level after MG132 treatment in the CPC samples but also detected in the SPI samples to some extent (Figure 4B), indicating that polyubiquitinated PhS_3L_-RNases are degraded in the CPC pollen tubes. Style extracts and immunoprecipitation of the untreated pollen tube samples did not show any signal as negative controls (Figure 4B). Taken together, these results showed that PhS_3L_-RNases are indeed polyubiquitinated in the CPC pollen tubes in the *in vitro* germination system, similar to that found *in vivo* (Figure 4A). In general, proteins with long polyubiquitin chains (at least four ubiquitin monomers) are recognized and degraded by the UPS (Thrower et al., 2000; Weissman, 2001). Thus, the increase of polyubiquitinated PhS_3L_-RNases after MG132 treatment suggested that the UPS pathway is involved in S-RNase degradation of the CPC pollen tubes.

### S-RNase degradation by the UPS pathway in the cross-compatible pollen tubes is mediated via SCF^SLF^

**Figure 5:**
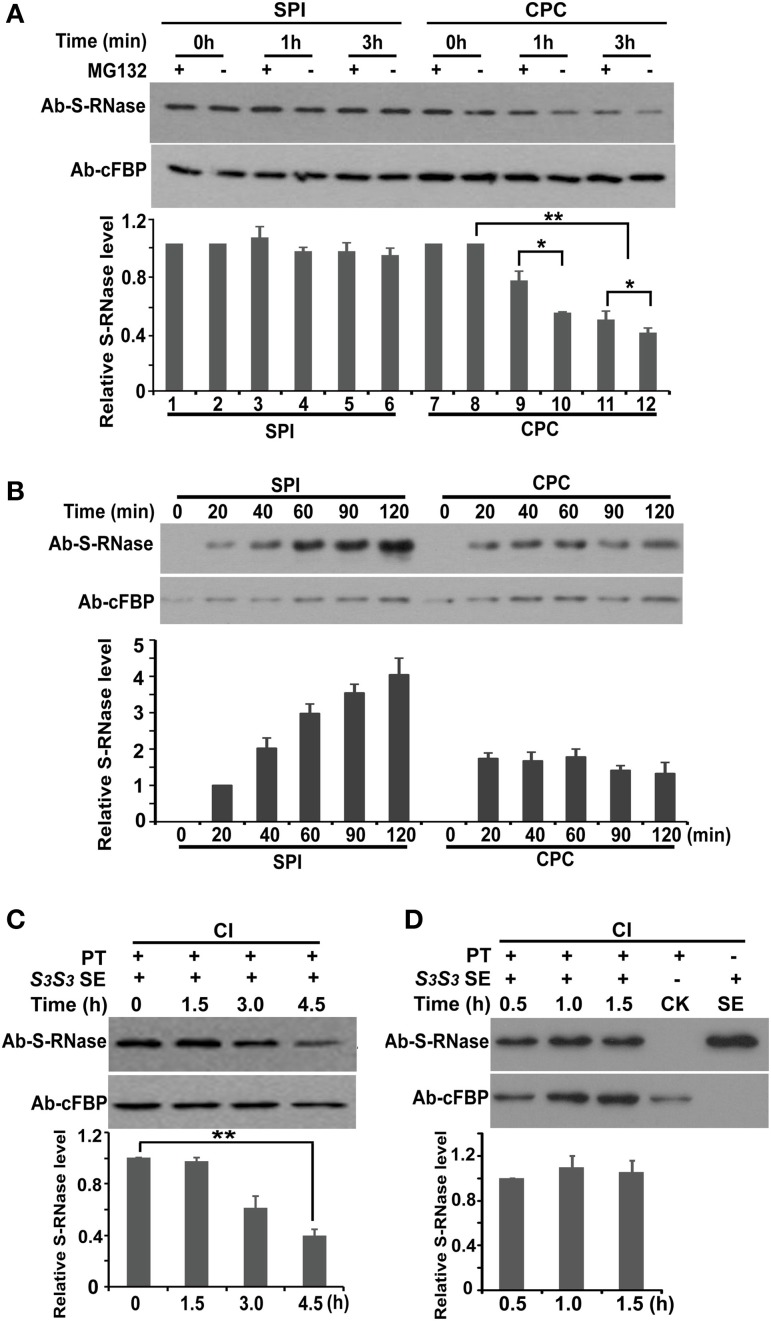
**SCF^SLF^ directly mediates non-self S-RNase degradation by the 26S proteasome pathway. (A)** Time course analysis of PhS_3L_-RNase levels in pulse challenged SPI (*S_3L_*) and CPC (*S_v_*) pollen tubes in conjunction with (+) or without (−) MG132 treatment. **(B)** Time course analysis of PhS_3L_-RNase accumulations in continuously challenged SPI (*S_3L_*) and CPC (*S_v_*) pollen tubes. *In vitro* germinated *S_3L_* and *S_v_* pollen tubes were continuously treated with *S_3L_S_3L_* style extract. PhS_3L_-RNase levels in pollen tubes were monitored by western blot analysis. cFBP was detected as loading control. **(C)** Time course analysis of PhS_3_-RNase levels in pulse challenged pollen tubes with stylar extracts. The pollen tubes were derived from the transgenic plants exhibiting competitive interaction (CI) between the transgene *PhS_3L_-SLF1* and *S_3_* haplotype. PhS_3_-RNase was detected by western blot to monitor its dynamics in *S_3_*/*PhS_3L_-SLF1* pollen tubes. **(D)** Time course analysis of PhS_3_-RNase levels in continuously challenged pollen tubes with stylar extracts. The transgenic *S_3_/PhS_3L_-SLF1* pollen tubes were continuously treated with *S_3_S_3_* style extract and monitored for S_3_-RNase level through the time course. CK indicates pollen tubes before treatment. cFBP was detected as loading control. PT, *S_3_/PhS_3L_-SLF1* pollen tube; SE, style extract. Column charts in both **(A–D)** show quantitative S-RNases levels determined by Quantity One software using three replicates. *T* tests were done between the designated lanes. Single and double asterisks denote significant and extremely significant difference, respectively.

The subcellular fractionation experiments showed that PhS_3L_-RNases were significantly reduced in the cytosol of the compatible pollen tubes, but remain essentially unchanged in SPI responses (Supplementary Figures S9A,B). Moreover, PhS_3L_-RNases were polyubiquitinated and their polyubiquitination levels increased after MG132 treatment in cross-compatible pollen tubes (Figures 4A,B). Thus, our results showed that PhS_3L_-RNases are degraded in the cross pollen tubes by the UPS pathway. Because of a high background expression level of PhS_3L_-RNase in style, it would be hard to directly examine whether PhS_3L_-RNases are degraded in the compatible pollen tubes *in vivo*. Instead, we carried out a pulse chase experiment using the *in vitro* germinated pollen tubes as described above. Both *S_3L_* (SPI) and *S_v_* (CPC) pollen tubes were challenged with *S_3L_S_3L_* style extracts and then rinsed and allowed to grow in fresh media in conjunction with or without MG132 treatment, respectively. Equal amounts of the SPI and CPC pollen tube samples were loaded and detected by PhS-RNase antibody. Western blot showed that PhS_3L_-RNase level was significantly decreased in CPC but still remained essentially unchanged in SPI response (Figure 5A), confirming that PhS_3L_-RNases are degraded in the CPC pollen tubes. In addition, MG132 treatment partially hindered the degradation of PhS_3L_-RNases in CPC but not in SPI response (Figure 5A), supporting the previous finding that PhS_3L_-RNase degradation occurs by the UPS pathway in cross-compatible pollen tubes.

To validate this finding, we further performed the following experiment also using the *in vitro* germinated pollen tubes. Both *S_3L_* (SPI) and *S_v_* (CPC) pollen tubes were continuously treated with *S_3L_S_3L_* style extracts, respectively. Equal amounts of the SPI and CPC pollen tube samples were loaded and detected by PhS-RNase antibody. Western blot showed that PhS_3L_-RNases were preferentially accumulated in incompatible pollen tubes, whereas PhS_3L_-RNase were not accumulated in compatible tubes when treated continuously with the style extracts (Figure 5B), showing that PhS_3L_-RNase is continuously degraded in cross-compatible pollen tubes.

To examine whether PhS_3L_-RNase degradation is mediated by SLF, we used the *S_3_S_3L_/PhS_3L_-SLF1* plants exhibiting breakdown of self-incompatibility described above (Supplementary Figures S3B, S4A and Supplementary Table S2) and carried out the following experiments. We first identified the *S_3_S_3_/PhS_3L_-SLF1* plants from the T_1_ progeny of *S_3_S_3L_/PhS_3L_-SLF1* and collected the pollen grains to perform the pulse chase experiment as described above. The *S_3_/PhS_3L_-SLF1* pollen tubes were pulse challenged with *S_3_S_3_* style extracts and then rinsed and left to grow in fresh media to monitor PhS_3_-RNase levels. Western blot analysis showed that PhS_3_-RNase levels in the *S_3_/PhS_3L_-SLF1* pollen tubes after being challenged with *S_3_S_3_* style extract were found to be gradually reduced (Figure 5C), similar to that in compatible pollen tubes (Figure 5A), showing that the degradation of PhS_3_-RNase is mediated by PhS_3L_-SLF1 action.

To validate this finding, we performed the following experiments. The *S_3_/PhS_3L_-SLF1* pollen tubes were continuously treated with *S_3_S_3_* style extracts. Equal amounts of the samples were loaded and detected by PhS-RNase antibody. Western blot analysis showed that PhS_3_-RNase was not accumulated in the continuously challenged *S_3_*/*PhS_3L_-SLF1* pollen tubes (Figure 5D), similar to that in compatible pollen tubes (Figure 5B), showing that S_3_-RNase is degraded continuously in the *S_3_/PhS_3L_-SLF1* pollen tubes. Taken together, these results suggested that S-RNase degradation in compatible pollen tubes is mediated by action of non-self SCF^SLF^.

## Discussion

Self and non-self-recognition of the pistil and pollen factors is one of the most intriguing processes of self-incompatibility. The pistil *S* determinant S-RNase activity has been shown to serve as cytotoxins to inhibit the pollen tube growth (McClure et al., 1990; Liu et al., 2009). Thus, compatible pollen tube must take an effective mechanism to restrict the S-RNase activity. Recently, two S-RNase restriction mechanisms, the S-RNase degradation or the S-RNase compartmentalization have been proposed to explain S-RNase fate during SI responses. In this study, we have provided several lines of evidence to support the S-RNase degradation mechanism. First, both immunogold labeling and subcellular fractionation showed that the two key pollen SI factors of *P. hybrida*, *PhS_3L_-SLF1* and PhSSK1, localize to the cytosols of both pollen grains and tubes and that the majority of S-RNase also has a similar localization after pollination, consistent with their joint roles in the pollen tubes. Second, both yeast two-hybrid and co-immunoprecipitation results showed PhSLFs and PhS-RNases directly interacts with each other, indicating that their recognition occurs in the cytosol where the initial self and non-self-signaling for the downstream discriminative responses could occur. Third, polyubiquitinated PhS-RNases were predominantly detected in the CPC pollen tubes both *in vivo* and *in vitro* by immunoprecipitation assay and their levels increased after MG132 treatment, consistent with the UPS pathway is involved in the polyubiquitinated S-RNase degradation (Zhang et al., 2009). Finally, we showed that the S-RNase degradation is mediated by action of non-self SCF^SLF^. Thus, our findings show that S-RNase degradation serves as the major mechanism for restricting S-RNase cytotoxicity in compatible pollen tubes. This conclusion is consistent with the most of the previous results (Takayama and Isogai, 2005; Zhang et al., 2009; Iwano and Takayama, 2012).

**Figure 6:**
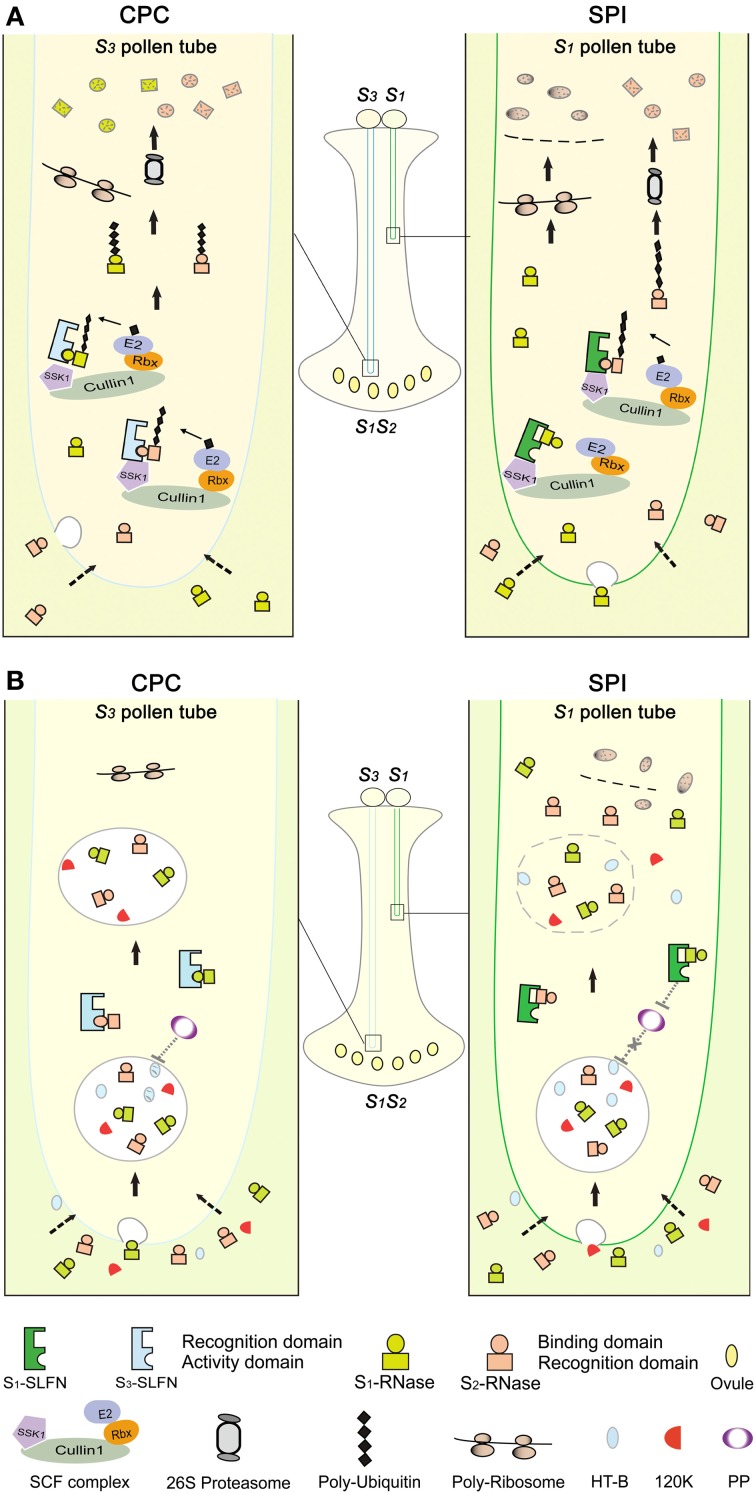
**Models of S-RNase-based self-incompatibility. (A)** S-RNase degradation model in *Petunia*. In CPC response, cross *S_3_* pollen lands on the stigma, germinates and grows into an *S_1_S_2_* style. Both S_1_- and S_2_-RNases enter the *S_3_* pollen tube by endocytosis or an unknown pathway (dotted arrows). Subsequently, the hypothetical binding domains of S_1_- and S_2_-RNases interact with a hypothetical activity domain of the pollen *S* (i.e., SLF in *Solanaceae* and *Plantaginaceae*) in the cytosol of the pollen tube. Then, one or more SLF (SLFN) in *S_3_* pollen tube form functional SCF^S3-SLFN^ complexes to tag S_1_- and S_2_-RNases with a polyubiquitin chain, which are subsequently degraded by the 26S proteasome. Thus, ribosomal RNAs escape from degradation by S-RNases in cross-pollen tubes, resulting in normal pollen tube growth. In SPI, during which self *S_1_* pollen lands on the *S_1_S_2_* style, both S_1_- and S_2_-RNases enter the *S_1_* pollen tube. Similar to CPC response, non-self S_2_-RNases bind to a hypothetical activity domain of the pollen *S* in the cytosol of the pollen tube. Then, one or more SLF (SLFN) in *S_1_* pollen tube form SCF^S1-SLFN^ complexes to tag S_2_-RNase with a polyubiquitin chain, resulting in its degradation by the 26S proteasome. In contrast, the recognition domain of self S_1_-RNase binds to a hypothetical recognition domain of SLF resulting in the formation of a non-functional SCF^S1-SLFN^ complex, thus self S-RNase escapes degradation and acts as a cytotoxin to inhibit the pollen tube growth. **(B)** S-RNase compartmentalization model in *Nicotiana*. S-RNases, 120K and HT-B all enter the pollen tubes. In CPC response, HT-B degradation possibly occurs by a hypothetical pollen protein (PP), thus S-RNase is sorted to a vacuolar compartment. In SPI, the interaction between S-RNase and SLF has been postulated to stabilize HT-B, perhaps by inhibiting the PP action. Subsequently, the vacuolar compartment breaks down, releasing S-RNase and acts as a cytotoxin to inhibit the pollen tube growth.

On the basis of our results and other recent findings, we propose a model for S-RNase-based self-incompatibility (Figure 6A). In this model, S-RNases are taken up into the cytosols of both CPC and SPI pollen tubes. In the cytosol, a repertoire of SLFs and S-RNases interact to trigger self or non-self-discriminative responses, respectively. Two hypothetical separate domains for SLF (recognition and activity domain) and S-RNase (recognition and binding domain) presumably mediate self and non-self-interaction between them as previously suggested (Kao and McCubbin, 1996). All SLF members in a given pollen tube form a spectrum of SCF^SLF^ complexes. In compatible pollen tubes, all SCF^SLF^ complexes could mediate the polyubiquitination of non-self S-RNases, which are destined to degradation via the UPS pathway. Whereas in incompatible pollen tubes, though non-self S-RNase is targeted for degradation similar to that in CPC, self S-RNase somehow escapes degradation by a yet unknown specific interaction between SLF and S-RNase, leaving it intact to exert a cytotoxic effect on self-pollen tubes and resulting in their growth inhibition.

Similar to previous models (Kao and Tsukamoto, 2004; Zhang et al., 2009; Chen et al., 2010), our model also predicts that S-RNases interacts with its self or non-self SLFs through different domains. But, it is not known where those domains are positioned in both S-RNases and SLFs. Our preliminary results suggest that the PhSLFs can selectively interact with “self” and “non-self” PhS-RNases in yeast system, and future studies will need to confirm these results *in vivo*. In addition, the “self” interaction between SLFs and S-RNases also have been detected in *Petunia inflata* (Solanaceae), *A. hispanicum* (Plantaginaceae) and apple (Rosaceae) (Qiao et al., 2004a; Hua and Kao, 2006; Yuan et al., 2014). Nevertheless, the interaction affinity analysis between PiS-RNase and PiSLF has shown that PiSLF interacts with non-self PiS-RNases more strongly than with self S-RNase and vice versa for SLFs in *Petunia inflata* (Hua et al., 2007). Recently, Kubo et al. found that a repertoire of *SLF* genes constituting the pollen *S* in a given *S*-haplotype and collectively control the pollen specificity against non-self S-RNases. They predicted that each type of SLF interacts with a subset of non-self S-RNases in a species and all SLF are required to detoxify all non-self S-RNases (Kubo et al., 2010). Thus, it is likely that a complicated relationship occurs among S-RNases and SLFs. In pollen tubes of a given *S*-haplotype, all of SLF proteins recognize and interact with self S-RNase but must through different domains from that with all non-self S-RNases. These differential interactions between S-RNase and self/non-self SLF might serve as an initial differential signaling mechanism for the downstream compatible and incompatible responses but further studies are needed to determine if this is the case *in vivo*.

The interactions between SLFs with non-self S-RNases have been shown to result in S-RNases polyubiquitination which is mediated by SCF^SLF^ complexes in our study, but it is not clear which lysine site(s) are ubiquitinated and whether monoubiquitination of S-RNases also occur. Other recent studies also showed that SCF^SLF^ complexes poly-ubiquitinate non-self S-RNases, resulting in their degradation *in vitro* (Entani et al., 2014; Yuan et al., 2014). Interestingly, some gold particles of PhS_3L_-RNase were also detected in the cytosol of the compatible pollen tubes even 24 h post pollination (Figure 2A), indicating that not all S-RNases are degraded in compatible pollen tubes. Recent studies also have shown that SI response requires the presence of a minimum level of S-RNase in the style, an amount referred to as a threshold level. A threshold value of S-RNases in the style is required for pollen rejection or acceptance (Qin et al., 2006; Soulard et al., 2014). Thus, in our results, S-RNase is likely degraded to a threshold value, but not all S-RNase would be degraded in cross pollen tubes. In addition, Qin et al. (2005) showed that replacing only one lysine residue in the C4 region (that is conserved in solanaceous S-RNases) with arginine did not affect SI function of S_11_-RNase in *S. chacoense*, indicating that this lysine is not involved in the S-RNase ubiquitination and degradation. In addition, F-box proteins themselves are known to be ubiquitinated and regulated by the UPS in mammals, yeast and plants (Galan and Peter, 1999; Wirbelauer et al., 2000; Stuttmann et al., 2009). Recently, Chen et al. (2012) found that a novel conserved ubiquitin-binding domain structure in the C-terminal regions of both PhS_3L_-SLF1 and PhS_1_-SLF1. Therefore, identification of potential ubiquitination sites and types of both S-RNases and SLFs is required to address those questions.

The S-RNase degradation model (Figure 6A) depicts of the S-RNase restriction mechanisms in compatible pollen tube, incompatible pollination, and competitive interaction. However, it is not entirely clear how the incompatible reaction occurs in pollen tubes. Recent studies have shown that S-RNases depolymerize actin cytoskeleton, trigger mitochondrial alteration and DNA degradation response in incompatible pollen tube, indicating that programmed cell death may occur in incompatible reaction of pears (*Pyrus pyrifolia*) (Liu et al., 2007; Wang et al., 2009; Wang and Zhang, 2011). In addition, studies have shown that HT-B protein, stylar 120 kDa glycoprotein and NaStEP are required for pollen rejection in *Nicotiana* but not for specificity (McClure et al., 1999; Hancock et al., 2005; Jimenez-Durán et al., 2013). Although these pistil factors have been identified, the underlying mechanisms for their action are currently unknown.

The S-RNase compartmentalization mechanism (Figure 6B) has been proposed for the S-RNase restriction mechanism during CPC responses in *Nicotiana*, but it remains unclear whether it functions in parallel, in sequence with the degradation mechanism. The two models make different presumptions about the interaction between S-RNases and SLFs. In the S-RNase degradation model, non-self S-RNase is degraded as a direct result of their interaction, indicating that SLFs are essential factors, consistent with genetic and molecular evidence of competitive interaction in both Solanaceae and Plantaginaceae (Golz et al., 1999; Xue et al., 2009; Sun and Kao, 2013). In the compartmentalization model, S-RNases are sequestered into a vacuole by a non-*S*-specific mechanism and an interaction between S-RNases and SLFs interferes with this process. In compatible pollinations, pollen overcomes rejection by degrading HT-B and compartmentalizing S-RNase, indicating that HT-B is essential factor. However, it is not clear how the S-RNase and SLF interaction controls HT-B degradation and vacuole membrane breakdown. Our results also appear to suggest that different mechanisms of the pollen reception are operating between *Nicotiana* and *Petunia*. It would be interesting to examine these potential differences by a comparative analysis using additional Solanaceaous species. Nevertheless, pollen tubes might possess a multilayered resistance mechanism against S-RNase where most non-self S-RNases are degraded to detoxify its activity but a small amount is compartmentalized and/or distributed in cytosol. In fact, our subcellular fractionation results showed that a minute amount of S-RNases are associated with a microsom-like fraction (**Figure S9A**). What role, in any, of the microsome-associated S-RNase plays in CPC and SPI responses remains to be determined.

In conclusion, our results showed that S-RNases are degraded in the cytosol of pollen tubes through the SCF^SLF^-mediated UPS pathway during the CPC response in *P. hybrida*. However, further studies including a detailed analysis of both S-RNase and SLF ubiquitinations and their types are urgently needed to address the biochemical mechanism of the SPI response and a potential link between the cytosolic and microsome-associated S-RNases during SPI and CPC responses.

## Materials and methods

### Plant materials and transformation

Wild-type self-incompatible homozygous *P. hybrida* lines (*S_3L_S_3L_*, *S_v_S_v_*, *S_1_S_1_*, and *S_3_S_3_*) have been previously described (Clark et al., 1990; Robbins et al., 2000; Qiao et al., 2004b). Heterozygous *S_3_S_3L_*, *S_3L_S_v_*, and *S_1_S_v_* were derived from crosses of related SI lines. Ti plasmid constructs were separately electroporated into *Agrobacterium tumefaciens* strain LBA4404 (Invitrogen), and transgenic plants were generated by Agrobacterium-mediated leaf disk transformation (Lee et al., 1994; Qiao et al., 2004b).

### Polymerase chain reaction (PCR)

The genomic DNA or cDNA were amplified by PCR using rTaq or ExTaq DNA Polymerase (Takara). Genotyping assays were carried out with genomic DNA. PCR primers are shown in Supplementary Table S8. They were used at a final concentration of 5 μM in 20 μl reactions. The PCR condition was 95°C for 5 min for hot start, then 35 cycles of the following: 95°C for 30 s, annealing at 52–58°C (about 3–5°C below the Tm of the primers used) for 30 s, extension at 72°C for 90 s, and finally incubated at 72°C for 10 min.

### Pollination

All of the pollinations were performed using open flowers. In the process of cross-pollination, anthers were removed from the flower of the plant serving as female recipient before dehiscence to prevent self-pollination. Pollinated flowers were covered with paper bags. Pollinated styles used in immunogold labeling, aniline blue staining and ubiquitination assay were performed as follows: self-pollinations were performed with wild-type *S_3L_S_3L_* plants and cross-pollinations with pollen from *S_v_S_v_* plants on *S_3L_S_3L_* plants.

### *In vitro* germination of pollen tubes

Mature pollen grains were suspended and incubated in liquid pollen germination medium (PGM, 20 mM MES, 15% PEG4000, 2% sucrose, 0.07% Ca(NO_3_)_2_·4H_2_O, 0.02% MgSO_4_·7H_2_O, 0.01% KNO_3_, 0.01% H_3_BO_3_, pH 6.0) at 25°C in the dark. For the treatment of pollen tubes, pollen tubes cultured for about 1.5 h were collected by centrifugation at 1000 g for 1 min and then suspended in fresh PGM with style lysates. For fractionation, immunogold labeling, co-immunoprecipitation, ubiquitination and S-RNase degradation assays, pollen tube samples were collected by centrifugation as above and then rinsed twice with fresh PGM to removal the S-RNases in PGM. Style lysates were prepared by grinding pistils to a fine powder and protein were extracted with PGM.

### Immunogold labeling and electron microscopy

Pollen grains, treated pollen tubes or pollinated styles (at least 2 or 3 replicates of each sample) were chemically fixed overnight at 25°C in 2.5% glutaraldehyde and 1.5% paraformaldehyde in 0.1 M PBS (pH 7.4). Samples fixed were rinsed several times with 0.1 M PBS and dehydrated sequentially with 30, 50, 70, 80, 90, 95, and 100% ethanol. Dehydrated samples were embedded in LR-White Resin (Sigma-Aldrich, USA). The embedded samples were sectioned into 90 nm sections with ultramicrotome (Leica EM UC6, German). The ultra-thin sections were placed on Formvar-coated nickel grids. Sections were blocked with PBS-Glycine buffer (PBS with 50 mM glycine) followed by PBGT buffer (PBS with 0.1% galtel, 1.0% BSA, 0.1% Tween 20) and incubated at 4°C overnight with primary antibody (1:2000 dilution of monoclonal anti-FLAG antibody for PhS_3L_-SLF1 labeling and 1:3000 dilution of polyclonal antibodies for PhSSK1 and PhS-RNase labeling, respectively). Polyclonal antibodies to PhSSK1 and PhS-RNase have been previously described (Zhao et al., 2010). Primary antibody labeled sections were washed with PBGT buffer 6 times and subjected to labeling with secondary antibody conjugated to 20 nm diameter colloidal gold particles at 1:20 dilution in PBGT buffer for 90 min at room temperature. After sequential washes with PBGT buffer, PBS-Glycine buffer (PBS with 50 mM glycine) and deionized water, twice for each wash, labeled sections were subjected to Danscher's enhancer solution at 25°C for 25 min to enlarge gold particles. Sections were stained with 2% uranyl acetate for 15 min and observed by using a HITACHI H-7500 transmission electron microscope (Danscher, 1981; Holgate et al., 1983).

### Subcellular fractionation and immunodetection

Pollen grains or *in vitro* germinated pollen tubes were homogenized with FastPrep-24 system (MP Biomedicals, USA) in homogenization buffer (HB) containing 330 mM sucrose, 150 mM KCl, 50 mM Tris-MES, pH 7.5, with additives of 1 mM EDTA, 1 mM PMSF, 1 mM DTT, and protease inhibitor cocktail. The fractionation procedure was performed on ice or at 4°C. The homogenate was subjected to differential centrifugation of three or four sequential steps with centrifuge force at 1000, 12,000, 40,000 g (omitted in three-step ones) and 160,000 g, respectively. The resulting pellets were suspended with HB buffer while the supernatant fraction was subjected to next step of centrifugation, before which an aliquot was stored for detection. Protein samples were quantified by the Bradford method. Equal amounts of protein samples were applied to 12% SDS-PAGE gels and transferred to PVDF membranes (GE Healthcare, USA) for western blot detection. The amounts of proteins were calculated from immuno signal determined by Quantity One software (Bio-Rad, USA). Antibodies used are as below, monoclonal antibody to the FLAG tag is a commercial product (Sigma-Aldrich, USA), polyclonal antibodies to organelle markers are all purchased (Agrisera, Sweden).

### Isolation and identification of PhS_3L_-RNase by LC-MS/MS

*S_3L_S_3L_* styles were extracted by trichloroacetic acid-acetone precipitation method. Proteins were dissolved from the dried precipitate using lysis-buffer (7 M urea, 2 M thiourea, 4% CHAPS, 65 mM DTT, 0.5% IPG buffer 3–10, 0.002% bromophenol blue.). Protein concentration was determined by the Bradford method. About 500 μg protein sample was loaded onto an IPG strip holder for a 24 cm, pH 3–10 non-linear gradient IPG strip (Bio-Rad) for the isoelectric focusing. For the SDS-PAGE, the equilibrated IPG gel strips were placed onto 12% gel. One gel was transferred to PVDF membranes for western blot detection. The other gel was visualized by Coomassie Brilliant Blue staining. The target protein was excised from the gel and digested with trypsin. Then the digested peptides identified by LC-MS/MS by Beijing protein innovation co., Ltd. All of the peptide fragments of the proteins were identified by the MASCOT server in-house search engine and compared against a merged database consisting of the Solanceae database and containing PhS_3L_-RNase. MASCOT search parameters were set as follows: threshold of the ions score cutoff, 0.05; peptide tolerance, 10 ppm; MS/MS tolerance, 0.2 Da; and peptide charge, 1+, 2+ or 3+. The search was also set to allow one missed cleavage by trypsin, carboxymethylation modification of Cys residues, and variable oxidation of Met residues.

### Aniline blue staining of pollen tubes within style

Pollinated styles were chemically fixed in ethanol: glacial acetic acid (3:1) solution 24 h or more at 25°C. Then, rinsing three times in water, pistils were transferred to 8 N sodium hydroxide solution for 8 h to clear and soften the tissue. After rinsing in water, the softened pistils were soaked in a 0.1% solution of water-soluble aniline blue dye dissolved in 0.1 N K_3_PO_4_ for 8–24 h in the dark at room temperature. The pollen tubes in the styles are smeared or are observed whole under a conventional or dissecting microscope by direct illumination with ultraviolet light of Olympus BX53 fluorescence microscope. Observations are made in a darkened room.

### Yeast two-hybrid assays

The full-length *PhS-RNases* were cloned into *pGADT7* (Clontech, CA, USA), respectively, to produce fusion proteins with the GAL4 activation domain. The C-terminal of *PhSLF*s(removing N-terminal approximately 60 amino acid long F-box motif) was introduced into *pGBKT7* (Clontech, CA, USA), to form recombinants with the GAL4 DNA binding domain. The various combinations of BD and AD vectors were co-transformed into yeast strain AH109 and grown on SD/-Leu-Trp medium at 30°C for 3 days. The clones were subsequently grown on SD/-Ade-His-Leu-Trp medium at 30°C for 7 days to test interaction. The Combinations of *AD::PhS-RNases* with empty BD are used for detecting PhS-RNases self-activation. The combinations of *BD::PhSLF* with empty AD were carried out to evaluate PhSLFs self-activation in yeast two-hybrid assays. The combinations of empty AD with empty BD indicate negative controls. For β-galactosidase assay, yeast clones grown for 48 h at 30°C were transferred onto filter paper, and the clones were lysed in liquid nitrogen, and then 5 mL Z buffer (60 mM Na_2_HPO_4_, 40 mM NaH_2_PO_4_, 10 mM KCl, and 1 mM MgSO_4_, pH 7.0) containing β-mercaptoethanol was added and incubated at 30°C (for <8 h) and checked periodically for the appearance of blue color.

### Co-immunoprecipitation

Mature pollen grains from transgenic plants of *S_1_/PhS_3L_-SLF1::FLAG* were suspended and incubated in liquid pollen germination medium described above. Total style proteins were extracted from *S_3_S_3_* and *S_3L_S_3L_* homozygous plants and co-incubated with germinated pollen tubes, respectively. For immunoprecipitation, anti-FLAG affinity gel (Sigma-Aldrich) was mixed with pollen tube extracts and co-incubated for 4 h at 4°C on a rotary shaker. After the affinity gel was washed with PBS buffer to remove all of the non-specific proteins. The protein being eluted from the resin can be monitored by measuring the absorbance of the eluant at 280 nm and further washed until the absorbance difference of the wash solution coming off the column is less than 0.05 vs. a wash solution blank as described procedure in ANTI-FLAG® M2 affinity gel (Sigma-Aldrich). Bound proteins were eluted with 0.1 M glycine HCl, pH 3.5. After equilibration of the eluted protein with 0.5 M Tris-HCl, pH 7.4, with 1.5 M NaCl, 2× SDS-PAGE sample loading buffer (125 mM Tris-HCl, pH 6.8, with 4% SDS, 20% glycerol, and 0.004% bromphenol blue) was added to each sample and control. The samples were denatured at 100°C for 5 min and then equal amount of protein samples were subjected to 12% SDS-PAGE gels and then transferred to PVDF membranes for western blot detection. Membranes were incubated with primary antibodies (1:3000 dilution of polyclonal antibody for PhS-RNase and 1:2000 dilution of monoclonal anti-FLAG antibody), and then incubated with anti-rabbit or anti-mouse IgG conjugated to horseradish peroxidase followed by chemiluminescence detection and then exposed to film.

### Ubiquitination assay

For the ubiquitination assay of *in vivo*, pollen tubes were dissected from pollinated styles. Pollinated styles were placed in extraction buffer (50 mM Tris-HCl, pH 7.5, 150 mM NaCl, 10 mM MgCl_2_, 1 mM EDTA, 1 mM PMSF, 0.1% NP-40, 10% glycerin and 1×protease inhibitor cocktail), then pollen tubes were carefully pulled out through a small slit in the style as described by Goldraij et al. (2006) in stereomicroscope and were frozen in liquid nitrogen. For the ubiquitination assay of *in vitro* pollen germination system, *S_3_L* and *S_v_* pollen tubes were continuously treated 1.5 h with *S_3L_S_3L_* style extract in conjunction with or without MG132 (40 μM), respectively, and then lysed in extraction buffer. Protein concentration was determined by the Bradford method. Protein extracts were incubated with primary antibody (polyclonal antibody for PhS-RNase) at 4°C for 4 h on a rotary shaker. Protein G agarose beads (IP50 Kit, Sigma-Aldrich, USA) were added and samples co-incubated for a further 2 h at 4°C on a rotary shaker. Samples were washed using IP buffer provided in the Kit. The protein/bead mixture was denatured at 100°C for 5 min and then equal amounts of protein samples were loading on 12% SDS-PAGE gels and then transferred to PVDF membranes for western blot detection. Membranes were incubated with primary antibodies (1:3000 dilution of polyclonal antibody for PhS-RNase and 1:2000 dilution of polyclonal antibody for ubiquitin), and then incubated with anti-rabbit IgG conjugated to horseradish peroxidase followed by chemiluminescence detection and then exposed to film.

### *In vitro* S-RNase degradation assays

For pulse challenged assay, *in vitro* germinated pollen tubes were co-incubated with style extracts for 1 h and then rinsed with style extracts and allowed pollen tubes to grow in fresh media to monitor S-RNase dynamics in pollen tubes. For continuously challenged assay, *in vitro* germinated pollen tubes were co-incubated with style extracts at different time points, and then lysed in extraction buffer (50 mM Tris-HCl, pH 7.5, 150 mM NaCl, 10 mM MgCl_2_, 1 mM EDTA, 1 mM PMSF, 0.1% NP-40, 10% glycerin and 1×protease inhibitor cocktail). Protein concentration was determined by the Bradford method. Equal amounts of protein samples were loaded on 12% SDS-PAGE gels and then transferred to PVDF membranes for western blot detection. Membranes were incubated with primary antibodies (1:3000 dilution of polyclonal antibody for PhS-RNase), and then incubated with anti-rabbit IgG conjugated to horseradish peroxidase followed by chemiluminescence detection and then exposed to film.

### Phylogenetic analysis

Analysis of *SLF* genes based on deduced amino acid sequences were carried out using a neighbor-joining method with 1000 bootstrap replicates using MEGA version 5.0 (Tamura et al., 2011). *PhS_1_-SLF1*, *PhS_3_A-SLF1*, *PhS_3_L-SLF1*, and *PhS_v_-SLF1* were cloned in this study; *S_17_* alleles are from *P. axillaris* (Tsukamoto et al., 2005) and all others from *P. hybrida* (Kubo et al., 2010).

## Author contributions

Yongbiao Xue conceived and designed the experiments. Wei Liu, Jiangbo Fan, Junhui Li, and Yanzhai Song performed the experiments. Qun Li and Yu'e Zhang provided technical support. Wei Liu and Yongbiao Xue were principally responsible for drafting the final manuscript. All authors provided intellectual content and contributed to manuscript revisions.

### Conflict of interest statement

The authors declare that the research was conducted in the absence of any commercial or financial relationships that could be construed as a potential conflict of interest.
