# Supplementary material for: SCFSLF-mediated cytosolic degradation of S-RNase is required for cross-pollen compatibility in S-RNase-based self-incompatibility in Petunia hybrida
Source: Front Genet. 2014 Jul 22;5:228. doi: 10.3389/fgene.2014.00228 (PMC4106197; doi:10.3389/fgene.2014.00228)
Supplement: Supplementary file 1 [file DataSheet1.DOCX]

**Supplementary Materials**

The supplementary materials contain eleven supplementary figures and eight supplementary tables.

**SCF^SLF^-mediated cytosolic degradation of S-RNase is required for cross-pollen compatibility in S-RNase-based self-incompatibility in *Petunia hybrida***

Wei Liu^1, 2*^, Jiangbo Fan^1, 2*^, Junhui Li^1, 2^, Yanzhai Song^1, 2^, Qun Li^1^, Yu’e Zhang^1^ and Yongbiao Xue^1**^

^1^ State Key Laboratory of Molecular Developmental Biology, Institute of Genetics and Developmental Biology, Chinese Academy of Sciences and National Center for Plant Gene Research, Beijing 100101, China

^2^ University of Chinese Academy of Sciences, Beijing 100190, China

^*^These authors contributed equally to this work

^**^Correspondence:

Yongbiao Xue

Institute of Genetics and Developmental Biology Chinese Academy of Sciences, 1 West Beichen Road, Chaoyang District, Beijing 100101, China

Tel: 86-10-62552880

Fax: 86-10-62537814

E-mail: ybxue@genetics.ac.cn

Running title: SCF^SLF^-mediated cytosolic degradation of S-RNase


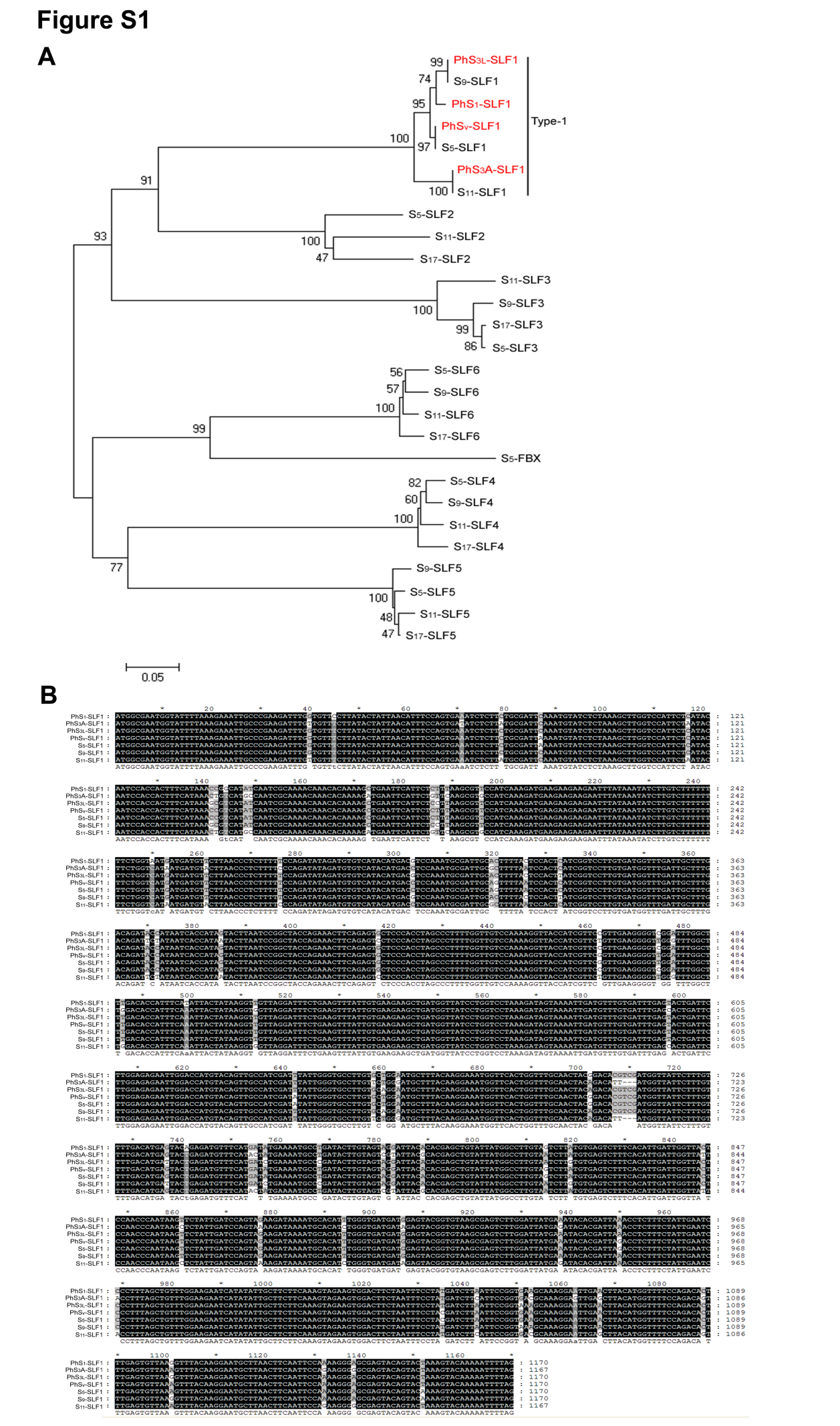


**Supplementary Figure S1.** Analysis of SLFs from *Petunia.* **(A)** The neighbor-joining tree of deduced amino acid sequences of *SLF* genes was created with 1,000 bootstrap replicates. *S_17_* alleles are from *P. axillar* (GenBank accession number: S_17_-SLF2, AB568397.1; S_17_-SLF3, AB568403.1; S_17_-SLF4, AB568409.1; S_17_-SLF5, AB568415.1; S_17_-SLF6, AB568421.1) and all others from *P. hybrida*. (GenBank accession number: PhS_1_-SLF1, GQ121443.1; PhS_3_A-SLF1, AY639403.1; PhS_3L_-SLF1, GQ121445.1; PhS_v_-SLF1, GQ121446.1; S_5_-SLF1, AB568390.1; S_9_-SLF1, AB568392.1; S_11_-SLF1, AB568393.1; S_5_-SLF2, AB568394.1; S_11_-SLF2, AB568396.1; S_5_-SLF3, AB568399.1; S_9_-SLF3, AB568401.1; S_11_-SLF3, AB568402.1; S_5_-SLF4, AB568405.1; S_9_-SLF4, AB568407.1; S_11_-SLF4, AB568408.1; S_5_-SLF5, AB568411.1; S_9_-SLF5, AB568413.1; S_11_-SLF5, AB568414.1; S_5_-SLF6, AB568417.1; S_9_-SLF6, AB568419.1; S_11_-SLF6, AB568420.1; S_5_-FBX, AB568423.1). **(B)** Alignment of nucleotide sequences of selected alleles of Type-1 *SLF* genes of *Petunia*. Nucleotide sequences of 100% similarity are shaded in black. Numbers show the positions of nucleotide.

**
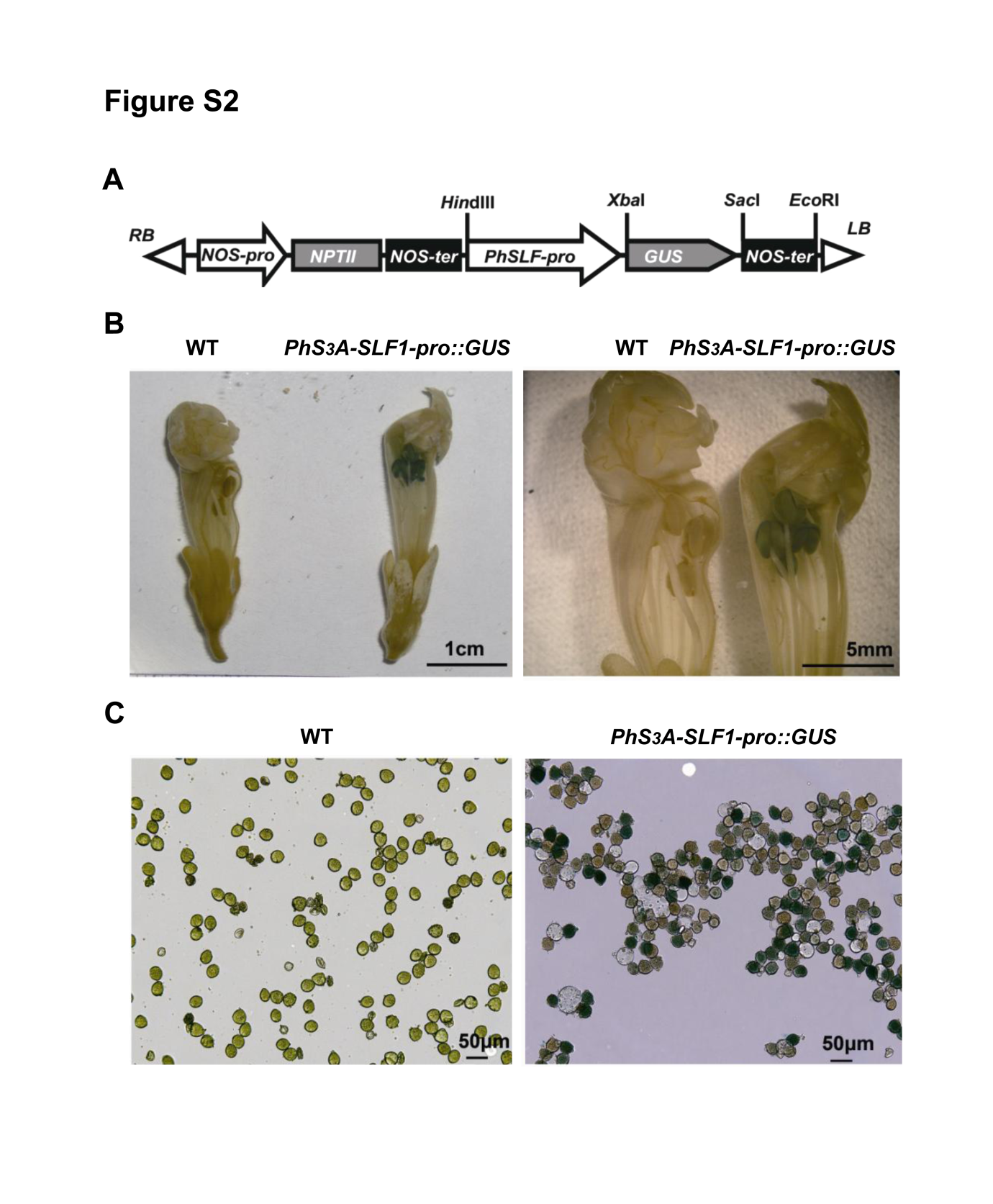
**

**Supplementary Figure S2.** Identification of a pollen-specific native promoter *PhS_3_A-SLF1 pro*. **(A)** A schematic representation of a binary vector containing the native *PhS_3_A**-SLF1* promoter. The 2120 bp sequence immediately upstream of the start codon of *PhS_3_A-SLF1* was inserted into *pBI101*, fused with downstream *GUS*, to determine its promoter activity. *RB*/*LB*, right border/left border of T-DNA; *NOS,* the gene coding for nopaline synthase*; pro*, promoter; *ter*, terminator; *NPTII*, the gene coding for neomycin phospho-transferase II conferring kanamycin resistance; *PhSLF-pro*, the candidate native pollen-specific promoter of *PhS_3_A-SLF1.* **(B)** Detection of GUS reporter expression in mature anthers of wild-type (WT) and the transgenic plant (*PhS_3_A-SLF1 pro::GUS*). **(C)** Detection of GUS reporter expression in pollen grains of wild-type (WT) and the transgenic plant (*PhS_3_A-SLF1 pro::GUS*).

**
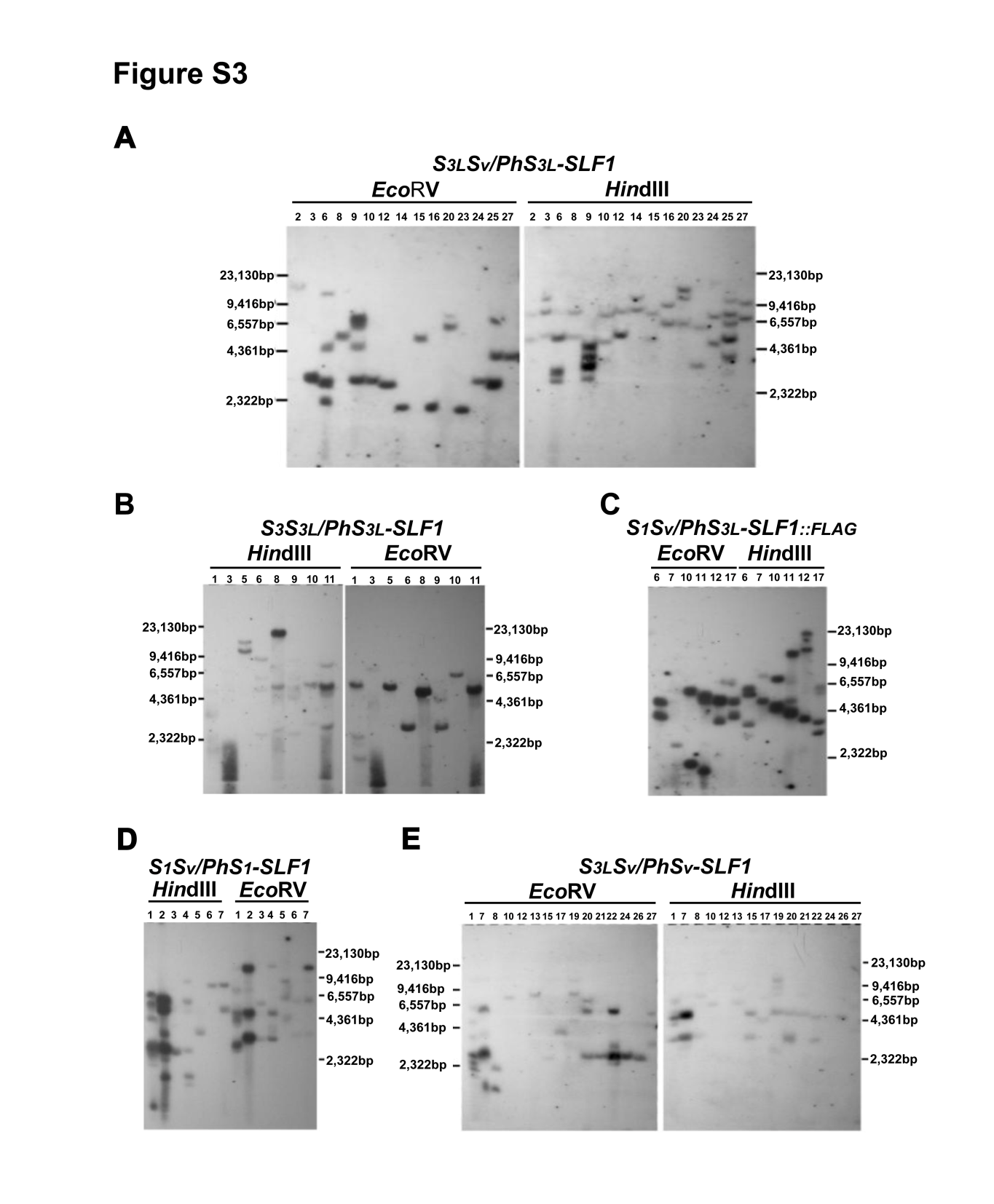
**

**Supplementary Figure S3.** Identification of primary *PhSLF*s transgenic lines by Southern blot. **(A)**-**(E)** Southern blot analyses of *S_3L_S_v_/PhS_3L_-SLF1* **(A)**, *S_3_S_3L_/PhS_3L_-SLF1* **(B)**, *S_1_S_v_/PhS_3L_-SLF1::FLAG* **(C)**, *S_1_S_v_/PhS_1_-SLF1* **(D)** and *S_3L_S_v_/PhS_v_-SLF1* **(E)** transgenic plants, respectively. Genomic DNA of the transgenic plants was separately digested by *Eco*RV and *Hin*d III and probed by the selective marker *NPTII*. The numbers labeled on the top of the blots denote the line identification numbers of the transgenic plants. Molecular weights in base pairs are shown on the right or left side of the blots.

**
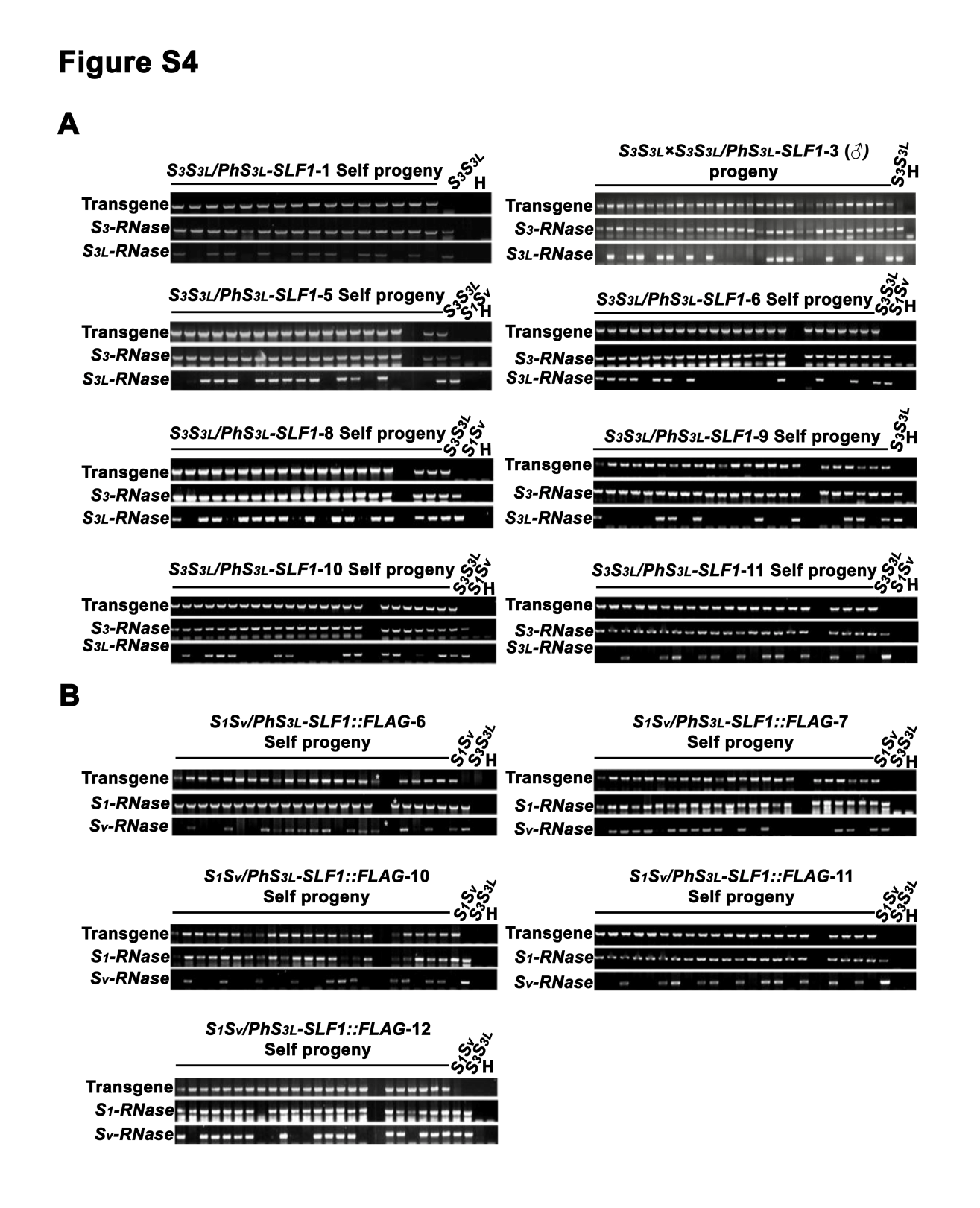
**

**Supplementary Figure S4.** PCR genotyping of T_1_ progeny from the primary *PhS_3L_-SLF1* transgenic plants. **(A)** Analysis of T_1_ progeny derived from the *S_3_S_3L_/PhS_3L_-SLF1* transgenic plants. A pair of *PhS_3L_-SLF1* forward primer and *NOS*-terminator reverse primer was used for PCR amplification of the *PhS_3L_-SLF1* transgenes. *S_3L_*-*RNase-* and *S_3_*-*RNase-*specific primer pairs were used to identify the corresponding *S* haplotypes. **(B)** Analysis of selfed T_1_ progeny derived from the *S_1_S_v_/PhS_3L_-SLF1::FLAG* transgenic plants. A pair of *PhS_3L_-SLF1* forward primer and *NOS*-terminator reverse primer was used for PCR amplification of the *PhS_3L_-SLF1::FLAG* transgenes. *S_1_*-*RNase-* and *S_v_*-*RNase-*specific primer pairs were used to identify the corresponding *S* haplotypes. The lines of *S_1_S_v_* and *S_3_S_3L_* were used as wild-type and H_2_O (H) as negative controls, respectively.

**
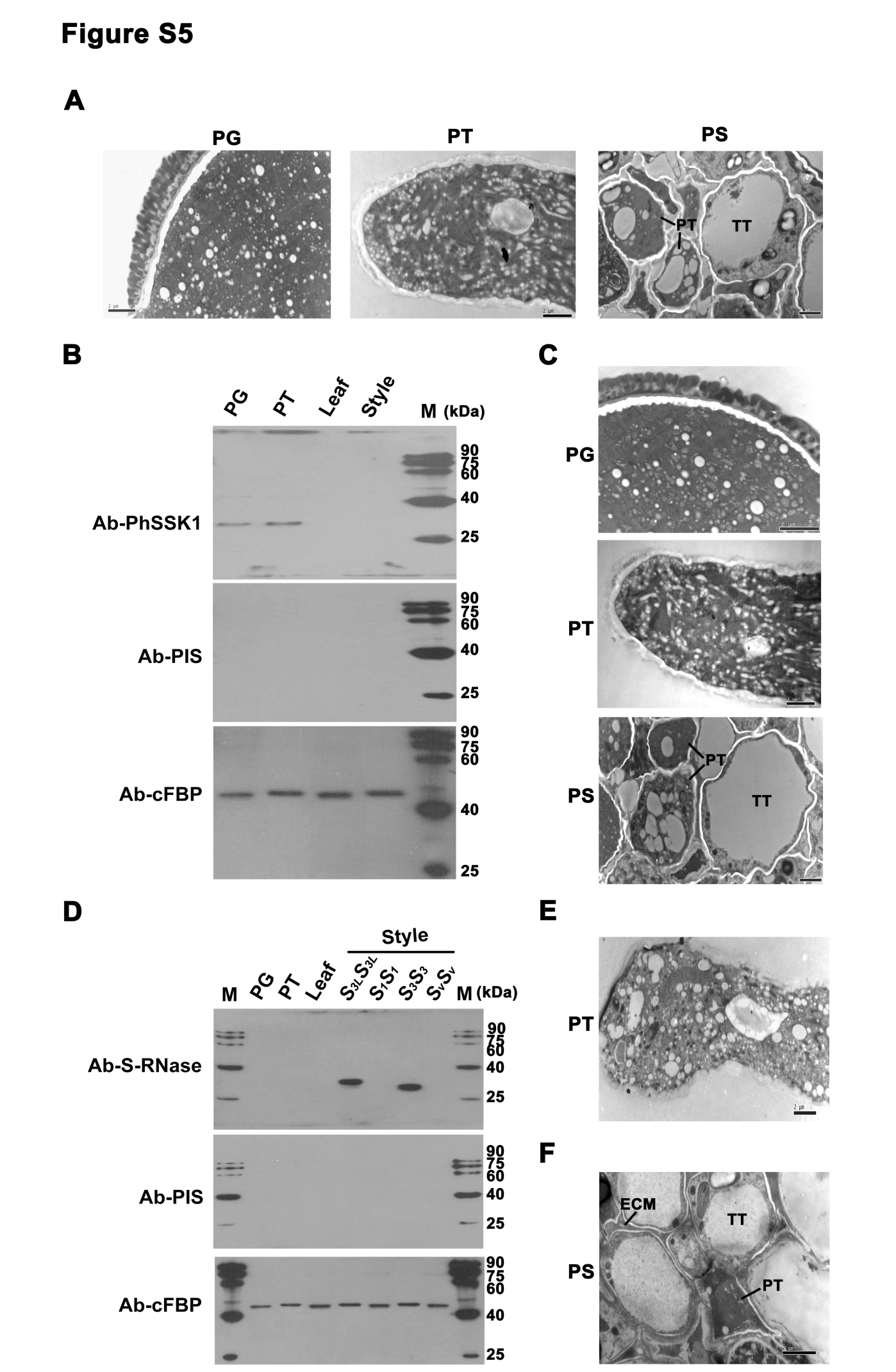
**

**Supplementary Figure S5.** Specificity analyses of FLAG, PhSSK1 and PhS-RNase antibodies. **(A)** Immunogold labeling detection of FLAG antibody using wild-type pollen grain (cross-sectioned), pollen tubes (longitudinal-section) and pollinated style (cross-sectioned). **(B)** Western blotting detection of antibody specificity of PhSSK1. Proteins were extracted from pollen grains, pollen tubes, leaves and pistils of *S_3L_S_3L_* plants. Rabbit pre-immune serum (PIS) was used as negative control and cFBP as loading control. Molecular weights in kilodalton (kDa) are shown on the right side of the blots. **(C)** Immunogold labeling detection of rabbit pre-immune serum as negative control for PhSSK1 antibody using wild-type pollen grain (cross-sectioned), pollen tube (longitudinal-section) and pollinated style (cross-sectioned). **(D)** Western blot detection of antibody specificity of PhS-RNase. Proteins were extracted from pollen grains, pollen tubes and leaves of *S_3L_S_3L_* plants, and style proteins were extracted from *S_3L_S_3L_*, *S_1_S_1_*, *S_3_S_3_* and *S_v_S_v_* plants, respectively. Rabbit pre-immune serum (PIS) was used as negative control and cFBP as loading control. Molecular weights in kilodalton (kDa) are shown on the right side of the blots. **(E)** Immunogold labeling detection of the PhS-RNase antibody using pollen tube (longitudinal-section) without style extracts treatment as negative control. **(F)** Immunogold labeling detection of rabbit pre-immune serum (PIS) as negative control of PhS-RNase antibody using pollinated style (cross-sectioned). PG, pollen grain; PT, pollen tube; PS, pollinated style; TT, transmitting tract; ECM, extracellular matrix.

**
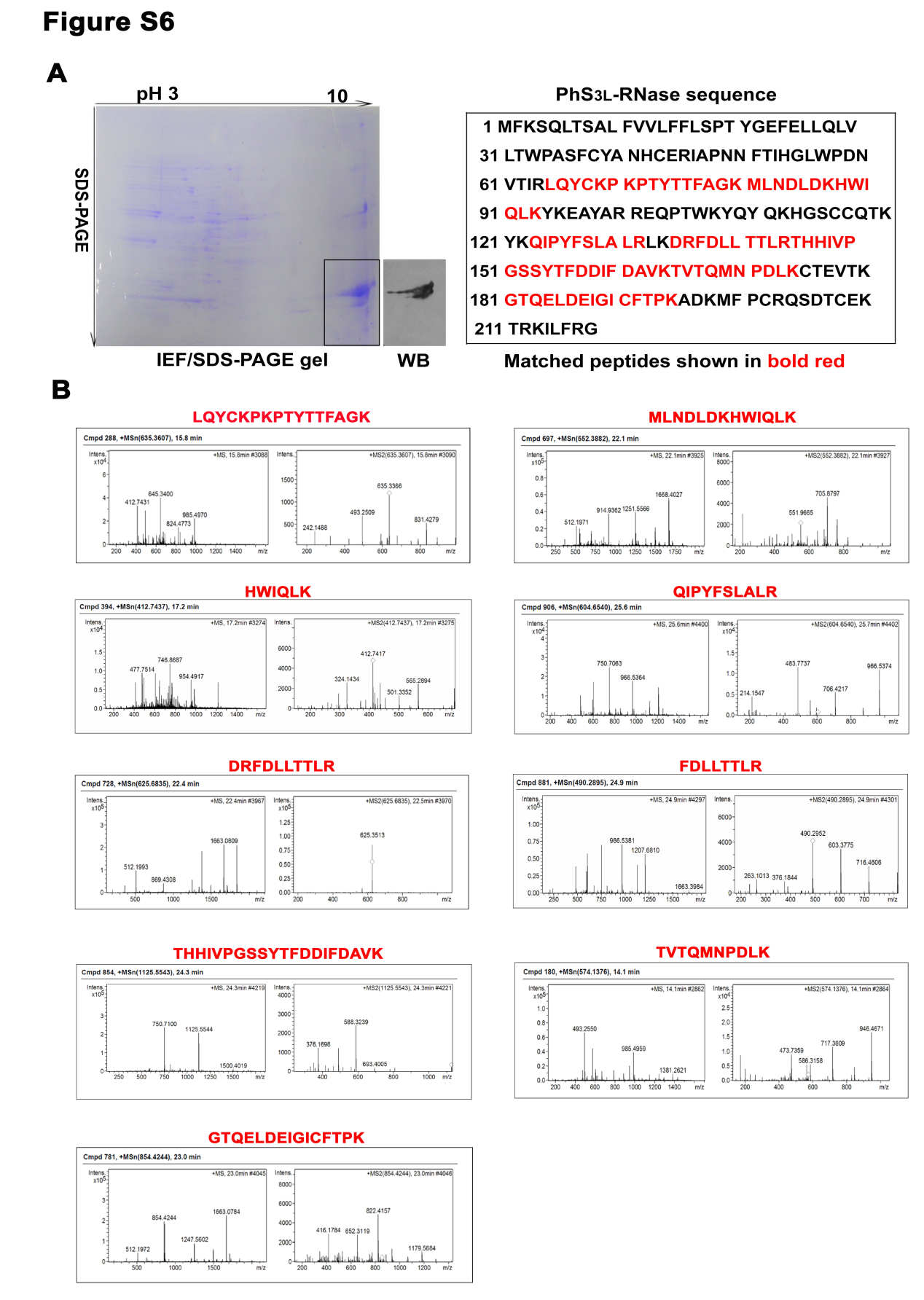
**

**Supplementary Figure S6.** PhS-RNase antibody specifically detects PhS_3L_-RNase. **(A)** *S_3L_S_3L_* style extracts were isolated and analyzed by two-dimensional polyacrylamide gel (IEF/SDS-PAGE), western blot (WB) and LC-MS/MS. **(B)** MS/MS spectra of nine matched peptides by LC-MS/MS of the target signal detected by western blot using PhS-RNase antibody.

**
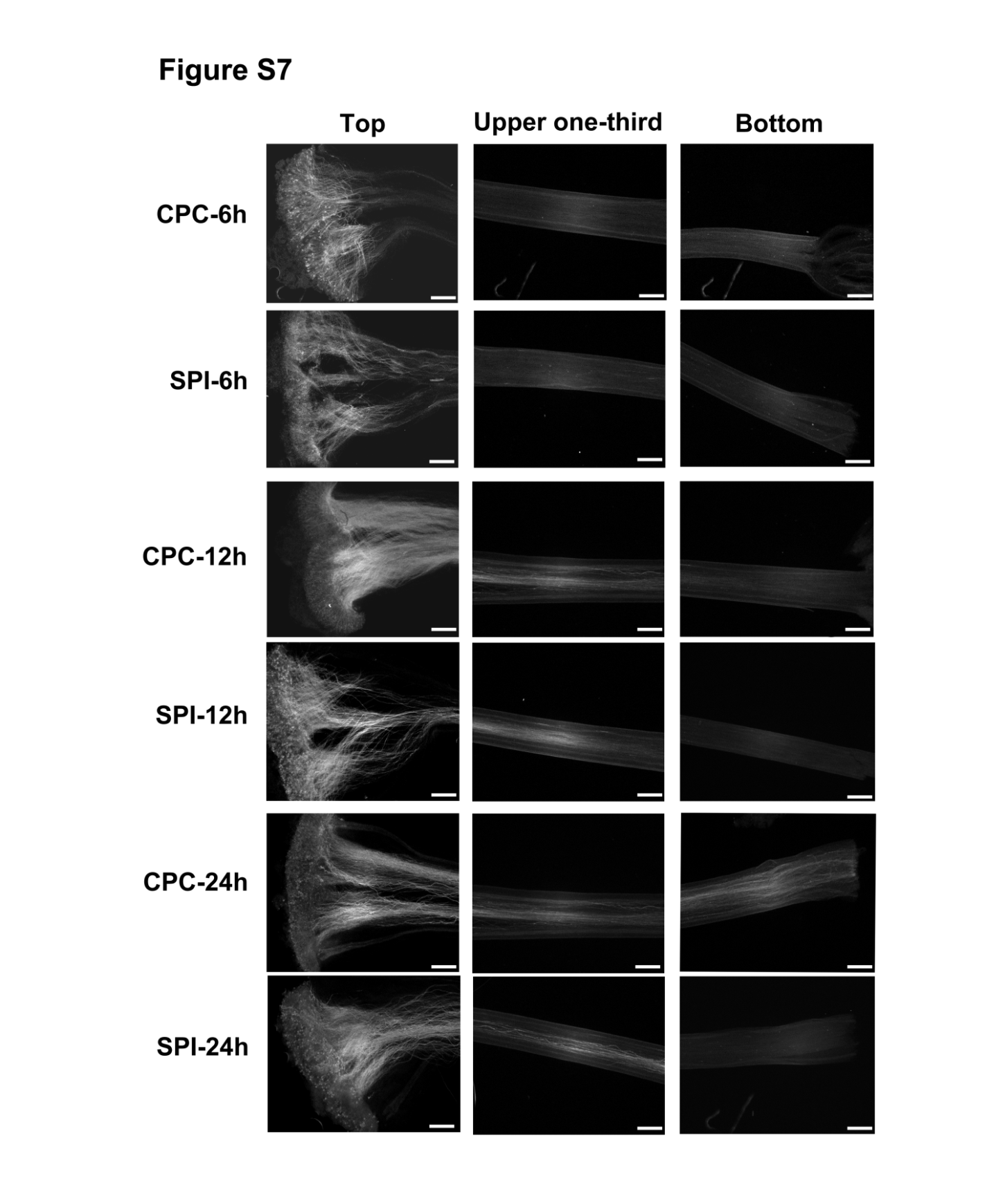
**

**Supplementary Figure S7.** Aniline blue staining analysis of pollen tube growth within pollinated styles. Compatible (CPC) and incompatible (SPI) styles after 6, 12 and 24 hours (h) post-pollination were stained with aniline blue and monitored by a fluorescence microscope. The top, upper one-third and bottom of pollinated styles were used to observe the pollen tube growth, respectively. Scale bar =100 μm.

**
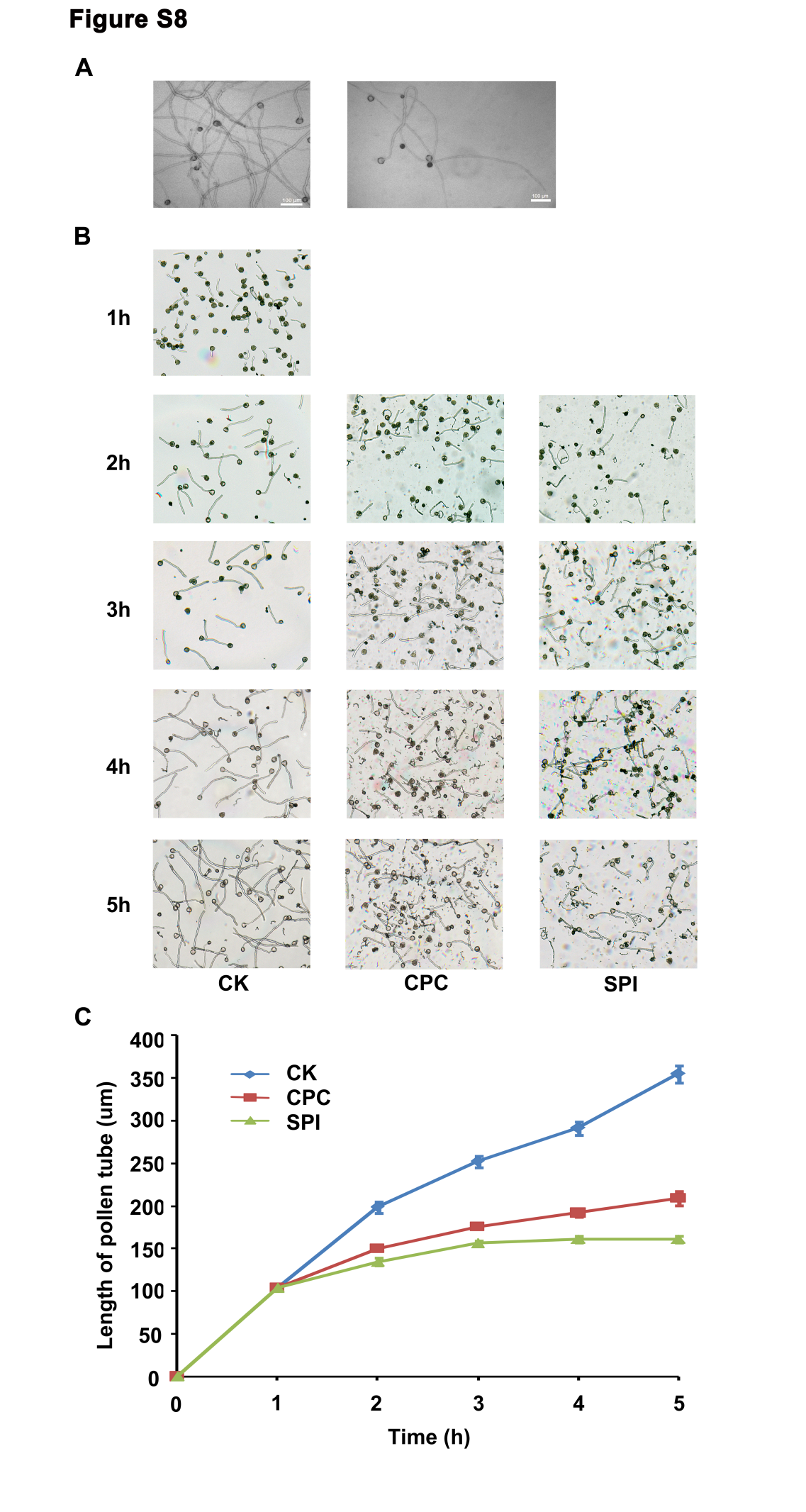
**

**Supplementary Figure S8.** *In vitro* pollen germination systems mimicking compatible and incompatible pollinations. **(A)** *S_v_* pollen tubes grow about 2 cm in *in vitro* pollen germination medium after 24 hours. **(B)** *S_v_* pollen tubes of untreated (CK) and treated with *S_3L_S_3L_* (CPC) and *S_v_S_v_* (SPI) style extracts at different time points. Scale bar = 100 μm. **(C)** Measurement of pollen tube length plotted *v.s.* time for *in vitro* germination experiments. Statistical data were calculated from samples of at least 50 individuals for every point. Error bars indicate S.E.M.

**
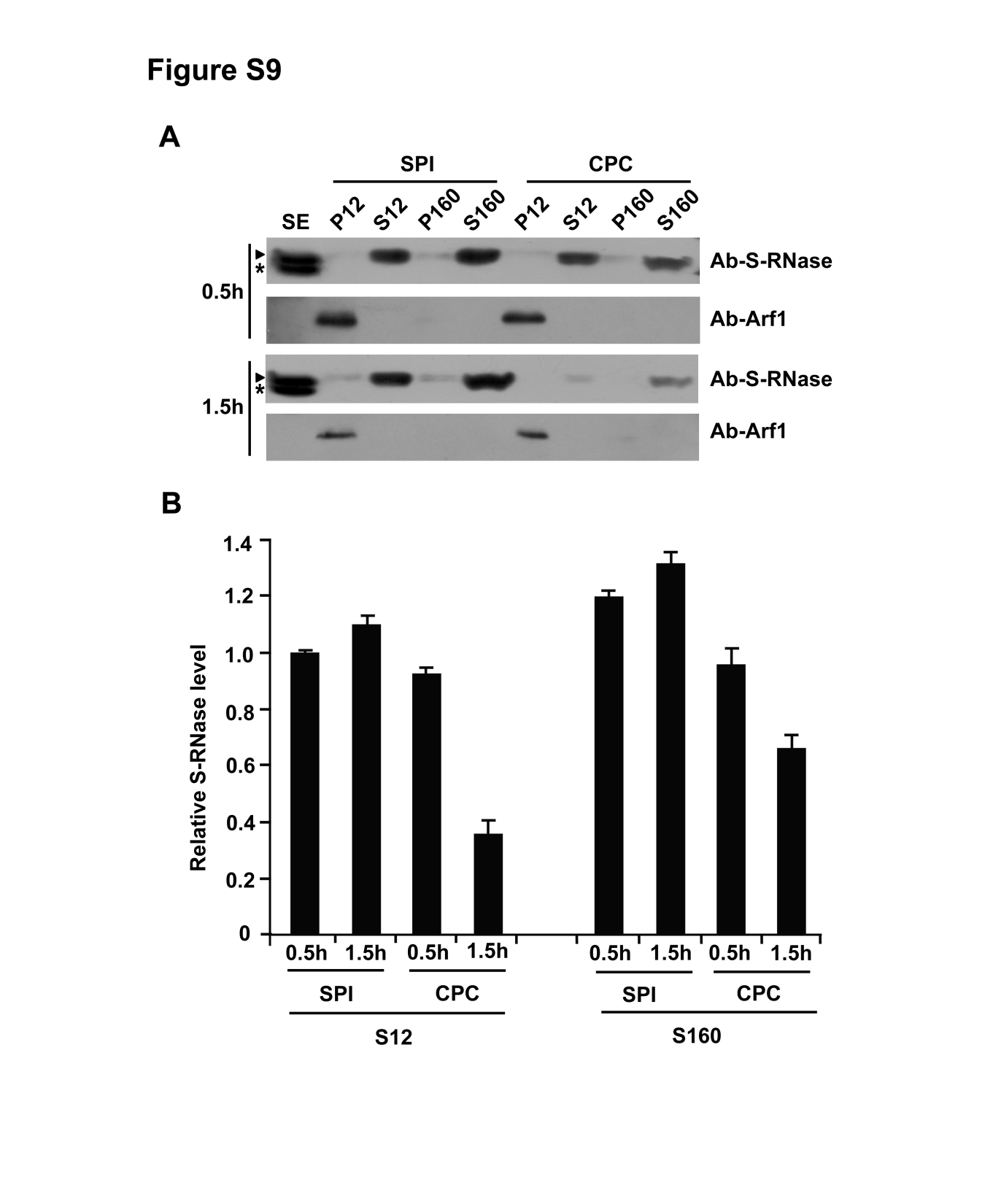
**

**Supplementary Figure S9.** S-RNases are significantly reduced in cytosol of compatible pollen tubes. **(A)** Western blot detection PhS_3L_-RNase in subcellular fractions of *in vitr*o germinated SPI (*S_3L_*) and CPC (*S_v_*) pollen tubes. Arf1 was detected to validate the fractionations. Pellet fractions (P12 and P160) and supernatant fractions (S12 and S160) are derived from differential centrifugation at 12,000 g and 160,000 g, respectively. **(B)** PhS_3L_-RNase levels within SPI and CPC pollen tubes determined by Quantity One software. The PhS_3L_-RNase levels were determined by using three replicates of the western blot. The black triangles in both **(A)** and **(B)** denote an N-glycosylated PhS_3L_-RNase form and the asterisks denote deglycosylated PhS_3L_-RNase form (Liu et al., unpublished data).


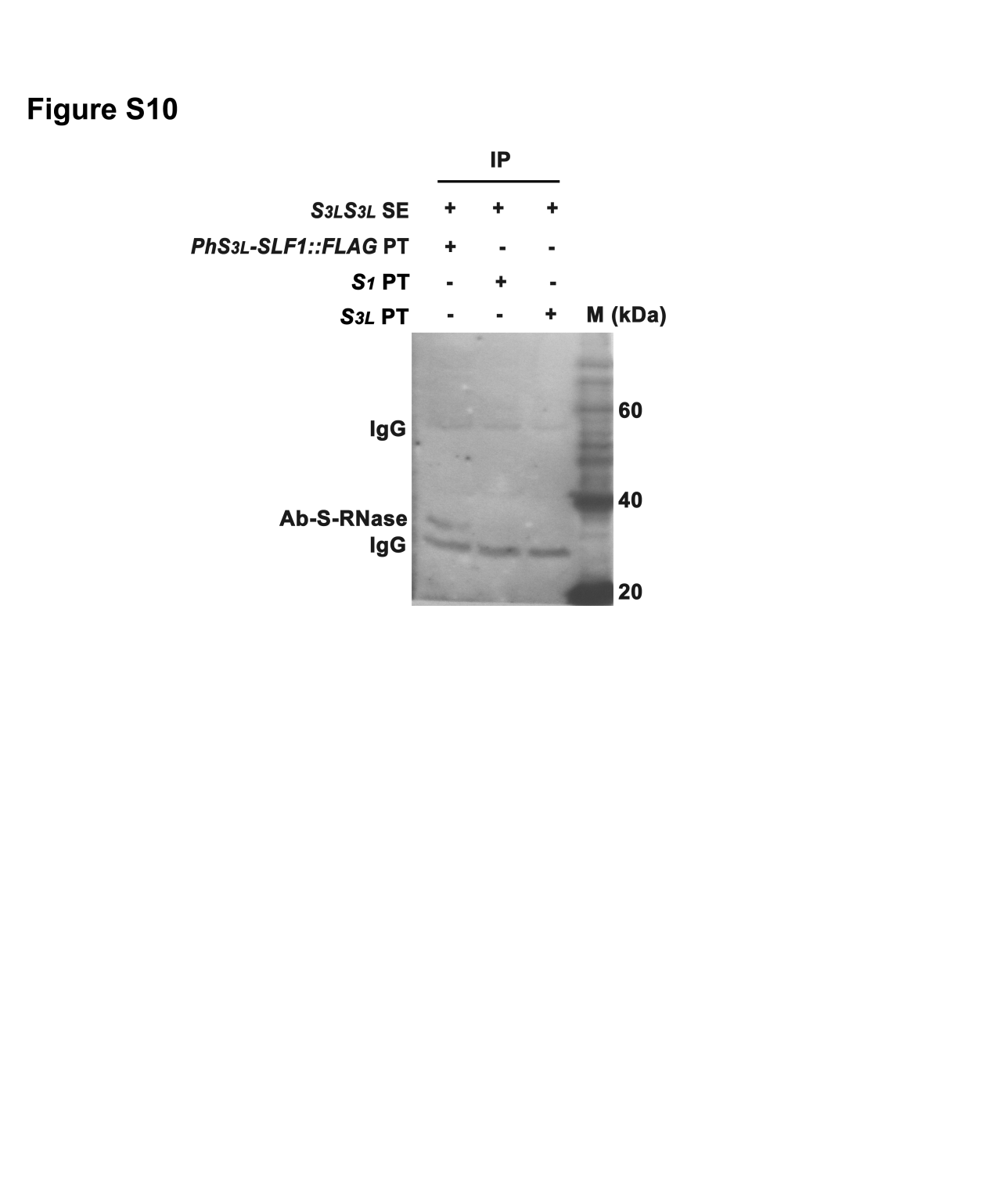


**Supplementary Figure S10.** Co-immunoprecipitation assays using of FLAG antibody. Pollen tubes from transgenic plants of *S_1_*/*PhS_3L_-SLF1::FLAG* were co-incubated with total style proteins were extracted from *S_3L_S_3L_* homozygous plants. *S_1_* and *S_3L_* pollen tubes from wild-type *S_1_S_1_* and *S_3L_S_3L_* plants were used as negative control. Samples were immunoprecipitated by FLAG antibody and detected by PhS-RNase antibody. Molecular weights in kilodalton (kDa) are shown on the right side of the blots. PT, pollen tube; SE, style extract; IgG, immunoglobulin G.

**
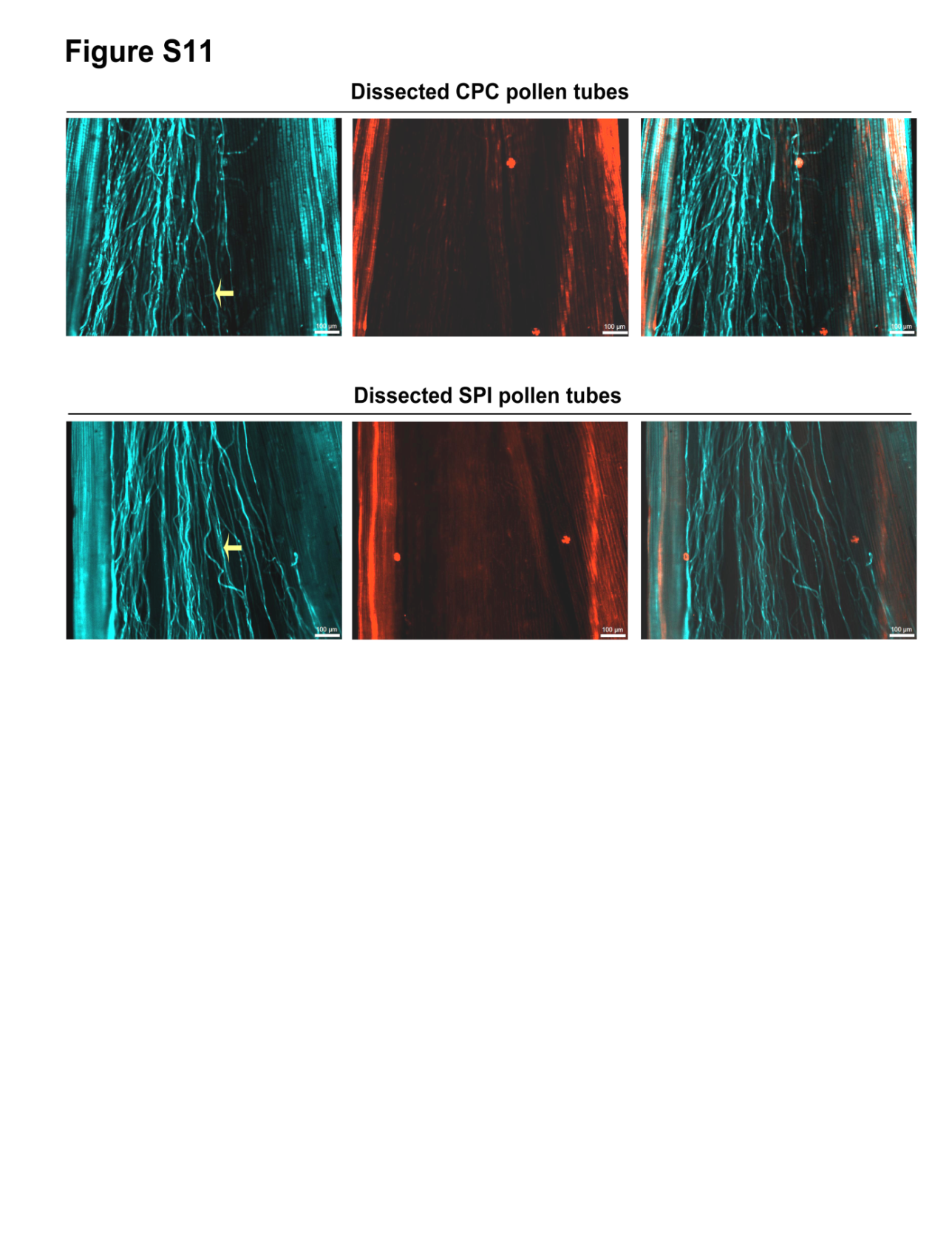
**

**Supplementary Figure S11.** Dissected pollen tubes from pollinated styles. Dissected CPC and SPI pollen tubes were stained with aniline blue and imaged through a ultraviolet filter to show pollen tubes (yellow arrow) on the left and through a Texas red filter to show the chlorophy ll fluorescence of transmitting tract cells (center), and the merged images are shown on the right. **Supplementary Table S1.** Results of self-pollination and the transgene expression of the primary *S_3L_S_v_*/*PhS_3L_-SLF1* transgenic plants.

| T_0_ plants | Self-pollination^a^ | Phenotype | Transgene expression^b^ |
| --- | --- | --- | --- |
| 2 | 0/17 | SI | + |
| 3 | 0/11 | SI | + |
| 6 | 0/15 | SI | + |
| 8 | 0/17 | SI | + |
| 9 | 0/18 | SI | + |
| 10 | 0/16 | SI | + |
| 12 | 0/16 | SI | + |
| 14 | 0/12 | SI | + |
| 15 | 0/15 | SI | + |
| 16 | 0/15 | SI | + |
| 20 | 0/11 | SI | + |
| 23 | 0/10 | SI | + |
| 24 | 0/14 | SI | + |
| 25 | 0/7 | SI | + |
| 27 | 0/13 | SI | + |

^a^ Data are represented as the number of capsules per number of pollinations.

^b^ Transgene expression was determined by RT-PCR.

**Supplementary Table S2:** Results of self-pollination of the primary *S_3_S_3L_*/*PhS_3L_-SLF1* transgenic plants and the S-RNase segregation of their progeny.

| T_0_ plants | Self pollination^a^ | Phenotype | Expected T_1_ ratio | Observed T_1_ ratio^b^ | χ^2^ test  χ^2^_0.01_=6.635, χ^2^_0.05_=3.841 |
| --- | --- | --- | --- | --- | --- |
| 1 | 12/12 | SC | 1:1 | 10:6 (16) | 1.00, P>0.05 |
| 2 | 10/10 | SC | 1:1 | n.t. | / |
| 3 | 15/15 | SC | 1:1 | 15:15 (30) | 0, P>0.05 |
| 4 | 11/11 | SC | 1:1 | n.t. | / |
| 5 | 11/11 | SC | 1:1 | 12:7 (19) | 1.32, P>0.05 |
| 6 | 16/16 | SC | 1:1 | 11:13 (24) | 0.17, P>0.05 |
| 7 | 14/14 | SC | 1:1 | n.t. | / |
| 8 | 12/12 | SC | 1:1 | 15:5 (20) | 5.00, 0.01<P<0.05 |
| 9 | 12/12 | SC | 1:1 | 9:14 (23) | 1.09, P>0.05 |
| 10 | 15/15 | SC | 1:1 | 13:11 (24) | 0.17, P>0.05 |
| 11 | 14/14 | SC | 1:1 | 11:10 (21) | 0.05, P>0.05 |

^a^ Data are represented as the number of capsules per number of pollinations.

^b^ Observed T_1_ ratios represent the number of the *S_3L_-RNase* progeny to that of the non-*S_3L_-RNase* progeny with the total number of T_1_ progeny analyzed in parentheses. n.t.: not tested.

**Supplementary Table S3:** Results of self-pollination of the primary *S_1_S_v_*/*PhS_3L_-SLF1::FLAG* transgenic plants and the S-RNase segregation of their progeny.

| T_0_ plants | Self pollination^a^ | Phenotype | Expected  T_1_ ratio | Observed  T_1_ ratio^b^ | χ^2^ test  χ^2^_0.01_=6.635, χ^2^_0.05_=3.841 |
| --- | --- | --- | --- | --- | --- |
| 1 | 6/6 | SC | 1:1 | n.t. | / |
| 2 | 7/7 | SC | 1:1 | n.t. | / |
| 3 | 5/5 | SC | 1:1 | n.t. | / |
| 5 | 4/4 | SC | 1:1 | n.t. | / |
| 6 | 5/5 | SC | 1:1 | 14:8 (22) | 1.64, P>0.05 |
| 7 | 2/2 | SC | 1:1 | 14:9 (23) | 1.09, P>0.05 |
| 9 | 3/3 | SC | 1:1 | n.t. | / |
| 10 | 4/4 | SC | 1:1 | 11:12 (23) | 0.04, P>0.05 |
| 11 | 10/10 | SC | 1:1 | 11:11 (22) | 0, P>0.05 |
| 12 | 6/6 | SC | 1:1 | 17:7 (24) | 4.17, 0.01<P<0.05 |
| 17 | 6/6 | SC | 1:1 | n.t. | / |

^a^ Data are represented as the number of capsules per number of pollinations.

^b^ Observed T_1_ ratios are the number of *S_v_-RNase* progeny to that of non-*S_v_-RNase* progeny with the total number of T_1_ progeny analyzed in parentheses.

n.t.: not tested.

**Supplementary Table S4:** Results of self-pollination and the transgene expression of the primary *S_1_S_v_*/*PhS_1_-SLF1* transgenic plants.

| T_0_ plants | Self-pollination^a^ | Phenotype | Transgene expression^b^ |
| --- | --- | --- | --- |
| 1 | 0/5 | SI | + |
| 2 | 0/6 | SI | + |
| 3 | 0/5 | SI | + |
| 4 | 0/7 | SI | + |
| 5 | 0/6 | SI | + |
| 6 | 0/10 | SI | + |
| 7 | 0/5 | SI | + |

^a^ Data are represented as the number of capsules per number of pollinations.

^b^ Transgene expression was determined by RT-PCR.

**Supplementary Table S5:** Results of self-pollination and the transgene expression of the primary *S_3L_S_v_*/*PhS_v_-SLF1* transgenic plants.

| T_0_ plants | Self-pollination^a^ | Phenotype | Transgene expression^b^ |
| --- | --- | --- | --- |
| 1 | 0/3 | SI | + |
| 7 | 0/2 | SI | + |
| 8 | 0/6 | SI | + |
| 10 | 0/4 | SI | + |
| 13 | 0/7 | SI | + |
| 15 | 0/8 | SI | + |
| 17 | 0/11 | SI | + |
| 19 | 0/10 | SI | + |
| 20 | 0/2 | SI | + |
| 21 | 0/3 | SI | + |
| 22 | 0/3 | SI | + |
| 24 | 0/4 | SI | + |
| 26 | 0/5 | SI | + |
| 27 | 0/4 | SI | + |

^a^ Data are represented as the number of capsules per number of pollinations.

^b^ Transgene expression was determined by RT-PCR.

**Supplementary Table S6:** Tryptic peptides identified from ca. 30 kDa protein spot.

| **1. S_3L_-RNase [*Petunia hybrida*] (calculated mass 26055, score = 6203)** | | | | | | |
| --- | --- | --- | --- | --- | --- | --- |
| Observed  mass | Experimental  mass^1^ | Calculated  mass^2^ | Error^3^ | Score | Peptide sequence |  |
| 412.7409 | 823.4672 | 823.4704 | 0.0033 | 39 | HWIQLK^4^ |  |
| 489.7842 | 977.5539 | 977.5546 | 0.0007 | 37 | FDLLTTLR^5^ |  |
| 573.7960 | 1145.5774 | 1145.5751 | 0.0023 | 78 | TVTQMNPDLQ |  |
| 581.7922 | 1161.5699 | 1161.5700 | 0.0001 | 69 | TVTQM^6^NPDLQ |  |
| 604.3448 | 1206.6750 | 1206.6761 | 0.0011 | 66 | QIPYFSLALR |  |
| 625.3525 | 1248.6904 | 1248.6826 | 0.0077 | 75 | DRFDLLTTLR |  |
| 551.9636 | 1652.8689 | 1652.8708 | 0.0020 | 55 | MLNDLDKHWIQLK |  |
| 557.3021 | 1668.8845 | 1668.8657 | 0.0188 | 53 | MLNDLDKHWIQLK |  |
| 854.4207 | 1706.8268 | 1706.8185 | 0.0083 | 97 | GTQELDEIGICFTPK |  |
| 635.0008 | 1901.9805 | 1901.9709 | 0.0095 | 31 | LQYCKPKPTYTTFAGK |  |
| 750.3736 | 2248.0990 | 2248.0801 | 0.0189 | 95 | THHIVPGSSYTFDDIFDAVK |  |

| **2. F-box protein At2g16365-like isoform 1 [*Solanum lycopersicum*] (calculated mass 28300, score = 22)** | | | | | | | |
| --- | --- | --- | --- | --- | --- | --- | --- |
| Observed  mass | Experimental  mass^1^ | Calculated  mass^2^ | Error^3^ | Score | | | Peptide sequence |
| 573.7966 | 1145.5786 | 1145.6656 | 0.0870 | 9 | | VTITGSVLTAGK | |
| 738.3938 | 1474.7730 | 1474.8355 | 0.0625 | 22 | NKVITGSLAGK | | |

| **3. Uncharacterized protein LOC101268822 [*Solanum lycopersicum*] (calculated mass 94537, score = 29)** | | | | | |
| --- | --- | --- | --- | --- | --- |
| Observed  mass | Experimental mass^1^ | Calculated mass^2^ | Error^3^ | Score | Peptide sequence |
| 459.7387 | 917.4628 | 917.4930 | 0.0303 | 29 | DGRGSLVSK |
| 623.9972 | 1868.9697 | 1868.9163 | 0.0534 | 2 | GTVDNTMRPEHLQISR |

| **4. Uncharacterized protein LOC101245704 [*Solanum lycopersicum*] (calculated mass 80197, score = 14)** | | | | | |
| --- | --- | --- | --- | --- | --- |
| Observed mass | Experimental mass^1^ | Calculated mass^2^ | Error^3^ | Score | Peptide sequence |
| 564.7865 | 1127.5584 | 1127.5458 | 0.0126 | 14 | DKTEEIEHK |

^1^*.* *m/z* transformed to a relative molecular mass from the observed mass.

^2^. Calculated relative molecular mass from the matched peptide sequence.

^3^. Difference between the calculated masses and experimental masses.

^4^. Peptide sequence also matched with S-RNase [*Solanum neorickii*].

^5^. Peptide sequence also matched with S_17_-RNase [*Solanum peruvianum*].

^6^. Underline indicates oxidized methionine residue.

**Supplementary Table S7:** Significant growth inhibition of SPI pollen tubes compared with CPC pollen tubes in *in vitro* pollen germination systems.

| Treatment | Pollen tube length (μm) | | | |
| --- | --- | --- | --- | --- |
|  | 2 h | 3 h | 4 h | 5 h |
| CPC | 149.986±3.46^a^ | 175.63±3.37 | 191.38±4.14 | 208.52±8.16 |
| SPI | 133.85±4.70 | 155.76±3.01 | 160.53±3.76 | 161.03±3.85 |
| P value | 0.005338 | 1.73E-05 | 10.5E-07 | 8.83E-08 |

^a^ Data represent average values ± SE, (n≥50);

P values are from Student’s t tests.

**Supplementary Table S8:** List of primer sequences shown from 5' to 3'.

| Primers | Genes name / Purpose | Sequences（5’ to 3’） |
| --- | --- | --- |
| *S_3L_R-F* | *S_3L_-RNase* | TGTGACGATCACTGAAATAAAT |
| *S_3L_R-R* | *S_3L_-RNase* | CGAAGAAAGGAATATGTTCATCC |
| *S_1_R-F* | *S_1_-RNase* | GTGAATTCTCTTTCGACCACTGGCAAC |
| *S_1_R-R* | *S_1_-RNase* | AGCTCGAGTCACTGTCGAAACGTAATCC |
| *S_v_R-F* | *S_v_-RNase* | GATTACGGACGAAGCTGATTG |
| *S_v_R-R* | *S_v_-RNase* | GAAACTTAATTATGGGTCCATAATCC |
| *S_3_R-F* | *S_3_-RNase* | CGAAAAGAAGCGTTTTCGTCTAG |
| *S_3_R-R* | *S_3_-RNase* | TCCGCACAGAGGTTTGTCAGTAC |
| *XbaI-SLF-F* | *PhSLF* construct | TCTAGAATGGCGAATGGTATTTTAAAG |
| *SacI-SLF-R* | *PhSLF* construct | CGAGCTCGCTGAAATTTATGTGCAAATTCG |
| *FALG-SacI-R* | *FLAG* tag | AACGAGCTCCTACTTGTCATCGTCGTCC |
| *SLF-KpnI-R* | *PhSLF-FLAG* construct | GGTACCAAATTTTTGTACTTTAGTACTGTAC |
| *pSLF-XbaI-R* | *Ph S_3_A-SLF1* promoter | TCTAGACCTTTATCCTCGAGAATGCACC |
| *SalI-pSLF-F* | *Ph S_3_A-SLF1* promoter | CGTCGACGGTTATAGCTTTTGTTATAAGCTT |
| *SLF-200-F* | *PhSLF* | GAGTACTGATTCTTGGAGAG |
| *NOS-UTR-R* | *NOS* terminator | TGCCAAATGTTTGAACGATC |

F: forward primer; R: reverse primer.
